# Circulating exosomal mRNA profiling identifies novel signatures for the detection of prostate cancer

**DOI:** 10.1186/s12943-021-01349-z

**Published:** 2021-03-30

**Authors:** Jin Ji, Rui Chen, Lin Zhao, Yalong Xu, Zhi Cao, Huan Xu, Xi Chen, Xiaolei Shi, Yasheng Zhu, Ji Lyu, Junfeng Jiang, Yue Wang, Tie Zhou, Jingyi He, Xuedong Wei, Jason Boyang Wu, Bo Yang, Fubo Wang

**Affiliations:** 1 Department of Urology, Shanghai Changhai Hospital, Naval Medical University (Second Military Medical University), Shanghai, China; 2 Division of Urology and Transplantation, Department of Surgery, Sichuan Academy of Medical Sciences and Sichuan Provincial People’s Hospital and Hospital of The University of Electronic Science and Technology of China, Chengdu, Sichuan China; 3 Research Center of Developmental Biology, Naval Medical University (Second Military Medical University), Shanghai, China; 4 Department of Urology, the First Affiliated Hospital of Soochow University, Suzhou, Jiangsu China; 5 Department of Pharmaceutical Sciences, College of Pharmacy and Pharmaceutical Sciences, Washington State University, Spokane, WA 99202 USA

**Keywords:** Exosome, Prostate cancer, RNA-sequencing, Diagnosis

## Abstract

The landscape and characteristics of circulating exosomal messenger RNAs (emRNAs) are poorly understood, which hampered the accurate detection of circulating emRNAs. Through comparing RNA sequencing data of circulating exosomes with the corresponding data in tissues, we illustrated the different characteristics of emRNAs compared to tissue mRNAs. We then developed an improved strategy for emRNA detection based on the features of circulating emRNAs. Using the optimized detection strategy, we further validated prostate cancer (PCa) associated emRNAs discovered by emRNA-seq in a large cohort of patients and identified emRNA signatures for PCa screening and diagnosis using logistic regression analysis. The receiver operating characteristic curve (ROC) analysis showed that the circulating emRNA-based screening signature yielded an area under the ROC curve (AUC) of 0.948 in distinguishing PCa patients from healthy controls. The circulating emRNA-based diagnostic signature also showed a great performance in predicting prostate biopsy results (AUC: 0.851). In conclusion, our study developed an optimized emRNA detection strategy and identified novel emRNA signatures for the detection of PCa.

**Supplementary Information:**

The online version contains supplementary material available at 10.1186/s12943-021-01349-z.

## Main text

Prostate cancer (PCa) is the leading malignancy in Western men, with 1,276,106 new cases and 358,989 deaths in 2018 globally [1]. The accurate early detection of PCa is one of the key issues in the management of PCa. The early diagnosis of PCa depends on prostate-specific antigen (PSA) test-initiated prostate biopsy. However, the wide use of PSA has resulted in a number of unnecessary biopsies accompanying complications due to its low specificity. In addition, a previous study showed that quite a few PCa cases, including high-grade PCa (HGPCa) cases, are missed since they can show PSA levels in the normal range [2]. Therefore, there is an urgent need to identify novel biomarkers with higher accuracy for the early detection of PCa.

Recent years have witnessed the promising roles of exosomal RNAs (exRNAs) in cancer detection [3]. Extracellular long RNAs (exLR), mainly messenger RNAs (mRNAs), could be potential biomarkers in glioma [4], hepatocellular carcinoma (HCC) [5], pancreatic ductal adenocarcinoma (PDAC) [6], etc. However, the landscape and characteristics of circulating exosomal mRNAs (emRNAs) are poorly understood [7], which hinders the accurate detection of emRNAs. Although there have been reports on the use of extracellular microRNAs [8, 9] and an extracellular three-gene panel [10, 11] to detect PCa and HGPCa early, no studies have focused on the diagnostic potential of circulating emRNAs. Here, we performed comprehensive sequencing of PCa-associated emRNAs, developed an optimized detection strategy for emRNAs and established novel emRNA-based signatures for the detection of PCa.

## The landscape and characteristics of circulating emRNAs

**Fig. 1:**
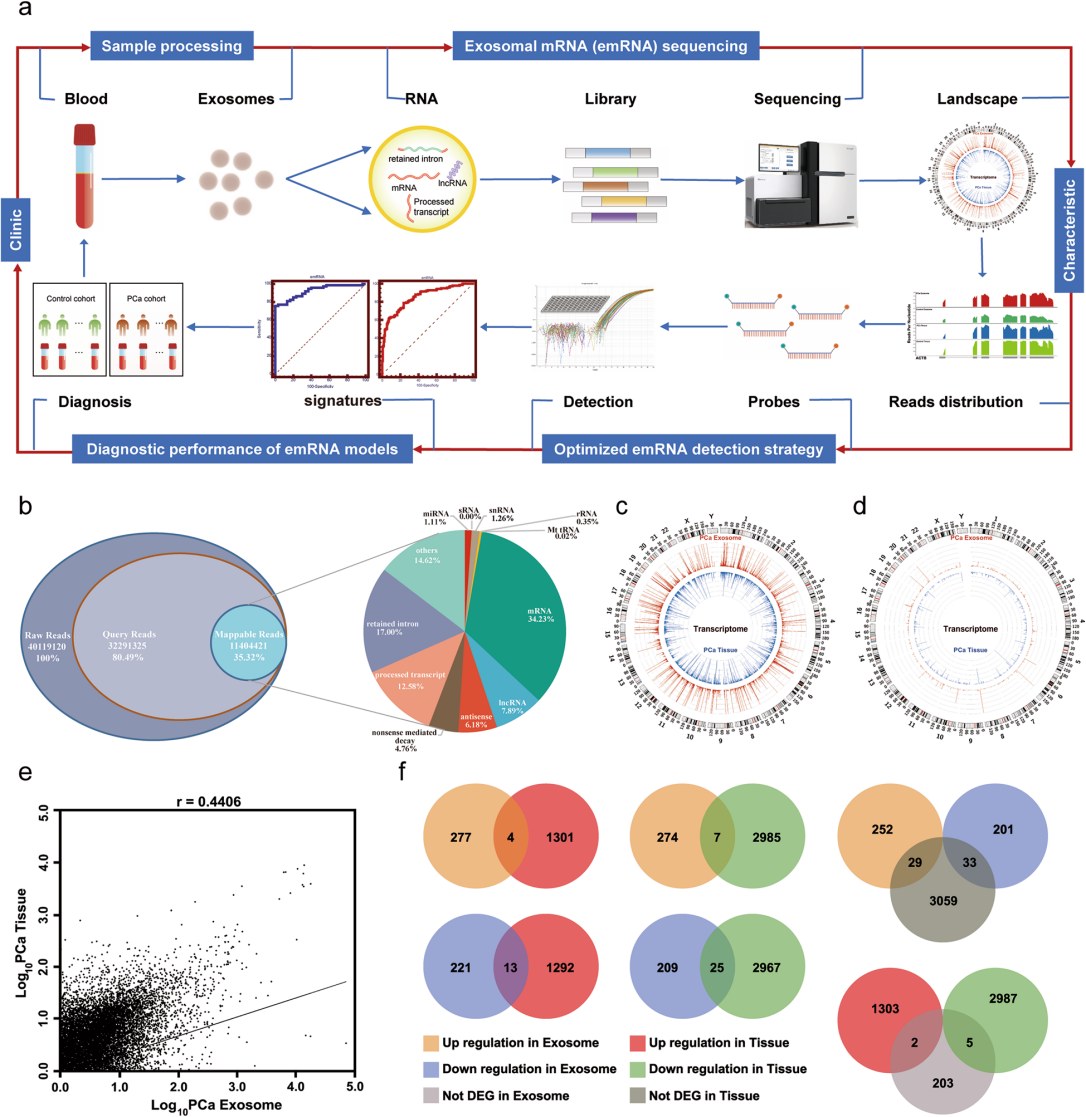
Characterization of circulating exosomal mRNAs (emRNAs). **a**, Workflow of the study, including sample processing, emRNA sequencing, demonstrating the landscape and characteristics of emRNA, optimizing the detection strategy, and identifying tumor-specific emRNA signatures. **b**, The type and distribution of RNAs in circulating exosomes. Raw reads are the sequences detected by RNA sequencing. Query reads are those after trimming. Mappable reads are those mapped to known human RNA or genomes. Circos plots showing all mRNAs (**c**), and oncogene mRNAs (**d**), from PCa tissues and circulating exosomes of the same cohort of patients. **e**, Scatter plot illustrating the correlation between tissue mRNA and emRNA levels. **f**, Venn diagrams showing the distinctive expression patterns between emRNAs and tissue mRNAs (based on the threshold of *p* value< 0.05 and fold change > 2 for upregulated and fold change < 0.5 for downregulated)

To better understand the potential role of emRNA in PCa detection, the landscape and characteristics of circulating emRNAs were first illustrated. The workflow of the study is summarized in Fig. 1a (see details of Study design in Additional file 1). The quality control of exosome isolation and verification is shown in Additional file 1: Figure S1. After analyzing the emRNA profiles in 31 PCa patients and 17 BPH (benign prostatic hyperplasia) individuals (negative prostate biopsy) by RNA-sequencing, we found that mRNA was the most abundant RNA, which was consistent with the findings of previous studies [5, 12] (Fig. 1b). We then compared the RNA sequencing data of circulating exosomes with the corresponding data in tissues. We mapped all mRNAs and mRNAs of oncogenes to the whole transcriptome of circulating exosomes and their corresponding tissues. The results indicated an overall even distribution of both mRNAs (Fig. 1c) and mRNAs of oncogenes (Fig. 1d) across the transcriptome between PCa tissues and exosomes. In addition, tissue mRNA levels were significantly correlated with emRNA levels (*r* = 0.441, Fig. 1e). These results showed that circulating emRNAs could reflect the tissue mRNA profiles, providing a promising noninvasive method for cancer diagnosis.

We further compared the expression levels of PCa-associated tissue mRNAs and circulating emRNAs. A total of 4 mRNAs were identified to be upregulated and 25 mRNAs were identified to be downregulated in both emRNAs and tissue mRNAs in PCa patients. Seven mRNAs were upregulated in circulating exosomes but downregulated in tissue, and 13 mRNAs were downregulated in circulating exosomes but upregulated in prostate tissue in PCa patients (Additional file 1: Table S1 and Fig. 1f). These results demonstrated that there were distinctive expression patterns between emRNAs and tissue mRNAs, making circulating emRNAs more attractive and usable as a noninvasive biomarker for PCa diagnosis.

## An improved strategy for emRNA detection

There is currently no consensus about the existing forms of emRNAs in peripheral blood, resulting in the inaccuracy and inconsistency of emRNA detection. A previous sequencing study estimated that most emRNAs appeared as shorter variants and fragments in the blood [5], which added to the difficulties in detection. Therefore, we first answered the question of whether circulating emRNAs were intact or presented as short variants before emRNA detection. We used Integrative Genomics Viewer (IGV) to visualize the reads distribution across the transcriptome of circulating emRNAs and tissue mRNAs. We compared the expression levels of each exon of candidate mRNA in circulating exosomes and in tissue and identified more variants in emRNAs than in tissue mRNAs (Additional file 1: Figure s2 and s4b-c, see details of Identification the existing forms of circulating emRNAs in Additional file 1). Therefore, we developed an optimized detection strategy for emRNAs as follows. First, we mapped the read density of each candidate mRNA across the transcriptome in circulating exosomes and in tissue to estimate the abundance of these variants. We designed multiple primers for different regions in the exons and validated the primers by reverse transcription polymerase chain reaction (RT-PCR) (Primers are listed in Additional file 1: Table S2). Then, the detectable and unique bands were chosen as the targeted amplicon of candidate emRNAs. Primers for RT-PCR and TaqMan probes for quantitative PCR (qPCR) were designed accordingly.

## EmRNA signatures for the detection of PCa

**Fig. 2:**
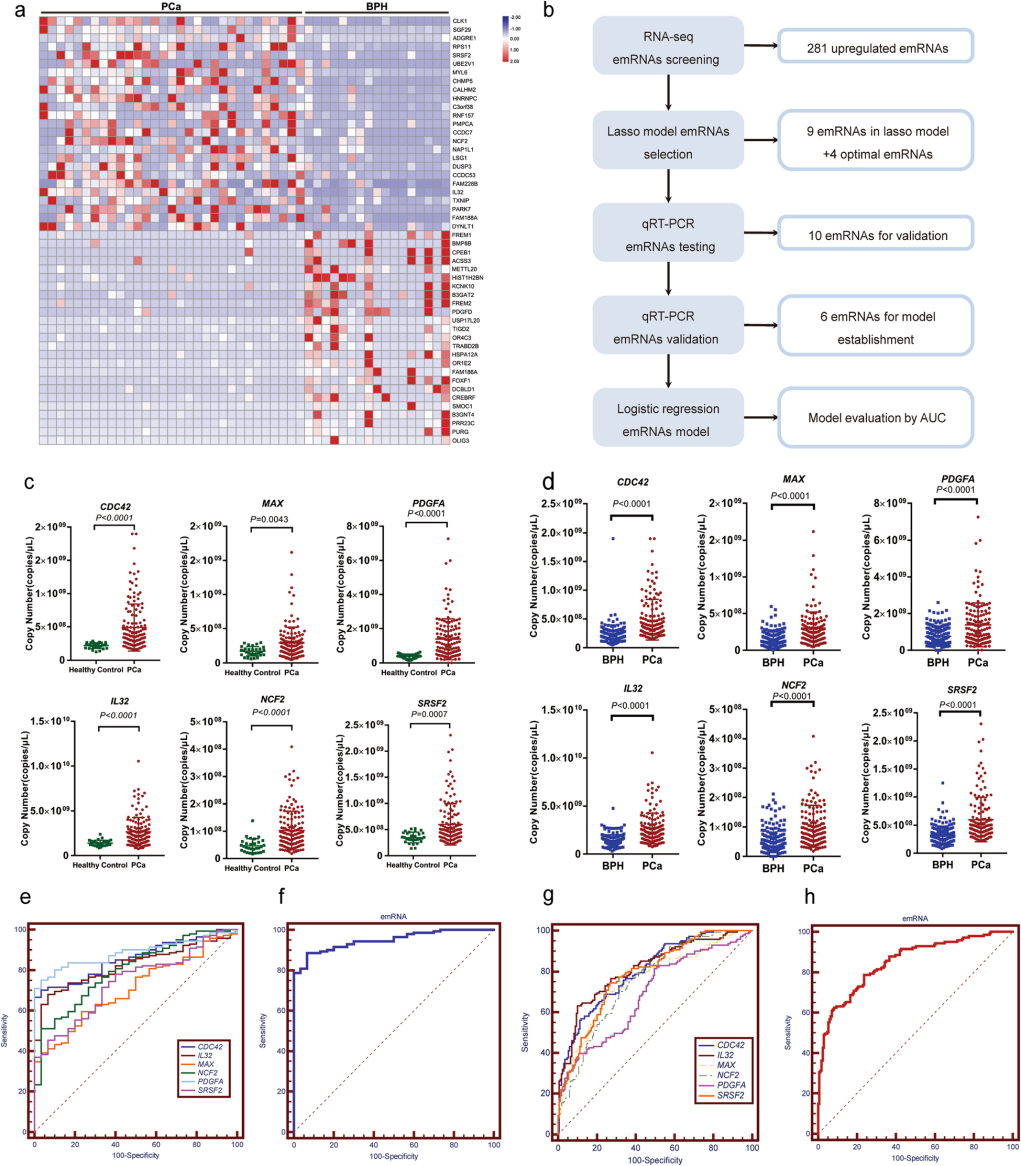
Validation of circulating exosomal mRNAs (emRNAs) as novel biomarkers for PCa diagnosis. **a**, Heatmap demonstrates the significantly dysregulated emRNAs in PCa patients. Each column represents an individual sample, and each row represents an emRNA. **b**, Workflow of the validation of potential circulating emRNAs. **c**, The scatter plot shows that the expression levels of circulating emRNAs, including *CDC42*, *IL32*, *MAX*, *NCF2*, *PDGFA* and *SRSF2*, are upregulated in PCa patients (*n* = 141) compared to healthy controls (*n* = 30). **d**, The scatter plot shows that the expression levels of circulating emRNAs, including *CDC42*, *IL32*, *MAX*, *NCF2*, *PDGFA* and *SRSF2*, are upregulated in PCa patients (*n* = 141) compared to patients with BPH (negative prostate biopsy, *n* = 170). **e** and **g,** ROC analysis shows the diagnostic performance of 6 mRNAs and the emRNA-based screening model (*CDC42*, *IL32*, *MAX*, *NCF2*, *PDGFA* and *SRSF2*; AUC: 0.948; *P* < 0.0001). **f** and **h,** ROC analysis shows the diagnostic performance of 6 emRNAs and the emRNA-based diagnostic model (*CDC42, IL32, MAX, NCF2, PDGFA and SRSF2*) (AUC: 0.851; *P* < 0.0001)

We identified PCa-associated emRNAs by RNA-sequencing. Representative upregulated (*p* < 0.05, fold change > 2) and downregulated (*p* < 0.05, fold change < 0.5) emRNAs are shown in Fig. 2a. We further evaluated the diagnostic performance of the PCa-associated emRNAs. The workflow is summarized in Fig. 2b. First, 281 upregulated emRNAs in PCa were identified (Additional file 1: Table S3). We then identified a total of 9 emRNAs (*TXK*, *ATM*, *TOX4*, *MAX*, *STK4*, *GRK5*, *PDGFA*, *RASSF5*, and *IL32*) with diagnostic potential for the detection of PCa by least absolute shrinkage and selection operator (LASSO) regression analysis (Additional file 1: Figure S3). Another 4 top upregulated emRNAs (*CDC42*, *FAM228B*, *NCF2* and *SRSF2*) according to the emRNA-seq results were also selected for further testing. The optimized detection strategy developed in this study was applied to evaluate the expression of the 13 emRNAs candidates (Additional file 1: Figure S4, see details of Optimized detection strategy for the detection of 13 PCa-associated emRNAs in Additional File 1). After testing in 10 pairs of PCa patients and controls, 3 emRNAs (*TXK*, *ATM* and *TOX4*) were excluded because they showed no difference between PCa patients and controls or showed inconsistencies with the RNA-seq results (Additional file 1: Figure S5a-f), and the remaining 10 detectable emRNAs (*MAX*, *STK4*, *GRK5*, *PDGFA*, *IL32*, *RASSF5*, *CDC42*, *FAM228B*, *NCF2* and *SRSF2*) were included for further validation. After evaluating the expression levels in 76 PCa patients and 84 BPH, 4 emRNAs (*STK4*, *GRK5*, *RASSF5* and *FAM228B*) were excluded because they showed no difference between PCa patients and controls **(**Additional file 1: Figure S5g-j). Finally, 6 upregulated emRNAs (*CDC42*, *IL32*, *MAX*, *NCF2*, *PDGFA* and *SRSF2*) were finally confirmed in 141 PCa patients, 170 BPH patients with negative prostate biopsy and 30 healthy controls. As shown in Fig. 2c, *CDC42*, *IL32*, *MAX*, *NCF2*, *PDGFA* and *SRSF2* were upregulated in PCa patients compared to healthy controls and achieved good performance for PCa screening (Fig. 2e). Furthermore, *CDC42*, *IL32*, *MAX*, *NCF2*, *PDGFA* and *SRSF2* were upregulated in PCa compared to BPH (negative prostate biopsy) (Fig. 2d) and achieved good performance for PCa diagnosis (Fig. 2g and Additional file 1: Table S4). We then used logistic regression analysis to establish an emRNA-based signature using the emRNAs described above. Receiver operating characteristic (ROC) analysis showed that the circulating emRNA-based screening signature (*CDC42*, *IL32*, *MAX, NCF2, PDGFA* and *SRSF2*) yielded an area under the ROC curve (AUC) of 0.948 in distinguishing PCa patients from healthy controls (Fig. 2f). More importantly, the circulating emRNA-based diagnostic signature (*CDC42*, *IL32*, *MAX*, *NCF2*, *PDGFA* and *SRSF2*) showed great performance in predicting prostatic biopsy results (AUC: 0.851) (Fig. 2h). We also established the subtype signatures based on clinical and molecular parameters for the detection of PCa (Additional file 1: Figure S8, see details of Established the subtype signatures for the detection of PCa and Correlation analysis between emRNAs and the grade of PCa aggressiveness in Additional file 1). Furthermore, the source and potential importance of circulating emRNAs was demonstrated (Additional file 1: Figure S9-10, see details of The source of circulating emRNAs and The potential importance of the emRNAs in Additional file 1). Our results indicated that emRNA-based signatures could serve as a novel and promising method for the detection of PCa.

## Conclusion

This is the first comprehensive study to investigate the characteristics of emRNA profiles in PCa patients and to evaluate the role of circulating emRNAs in the detection of PCa. In this study, we developed an optimized emRNA detection strategy and identified novel emRNA signatures for PCa screening and diagnosis. These signatures could serve as novel noninvasive biomarkers for the improvement of PCa diagnosis.

## Supplementary Information

**Additional file 1: Figure S1.** Quality control of exosomes isolation. **Figure S2**. Identificationc the existing forms of circulating emRNAs. **Figure S3.** Selection of potential diagnostic exosomes mRNA in the LASSO model. **Figure S4.** Optimized detection strategy for the detection of 13 PCa-associated emRNAs. **Figure S5.** Scatter plots of emRNA expression validation. **Figure S6.** Testing of previous reported reference genes. **Figure S7.** Standard curve generated with real-time quantitative PCR. **Figure S8.** Established the subtype signatures for the detection of PCa. **Figure S9.** EmRNAs are derived from PCa and then released into the cell culture medium or circulation by packing into exosomes. **Figure S10.** The potential biological function of the emRNAs. **Table S1.** The list of dysregulated transcripts with varied expression between tissue and serum exosomes. **Table S2.** The list of primers and probes. **Table S3.** The list of upregulated emRNA in PCa. **Table S4.** Diagnosis performance of emRNAs. **Table S5.** Demographics of PCa patients and control participants for QC of exosome isolation. **Table S6.** Demographics of PCa patients and control participants for RNA-seq of their serum exosome. **Table S7.** Demographcs of PCa patients and control participants for dysregulated emRNAs validation. **Table S8.** Demographics of PCa patients and control participants for TaqMan qPCR testing. **Table S9.** Diagnosis performance of emRNAs in different PSA group. **Table S10.** Diagnosis performance of emRNAs in different ages. **Table S11.** Diagnosis performance of emRNAs in differentiating BPH and PCa with GS 6 from PCa with GS ≥7. Identification the existing forms of circulating emRNAs. Optimized detection strategy for the detection of 13 PCa-associated emRNAs. Established the subtype signatures for the detection of PCa. Correlation analysis between emRNAs and the grade of PCa aggressiveness. The source of circulating emRNAs. The potential importance of the emRNAs. Methods.

## Data Availability

All data associated with this study are presented in the paper and the Additional File 1. Materials and the online databases are indicated in the Methods section of the Additional File 1. Correspondence and requests for materials should be addressed to F.W. (wangbofengye@163.com).

## Abbreviations

mRNA: Messenger RNA
emRNA: Exosomal messenger RNA
PCa: Prostate cancer
BPH: Benign prostatic hyperplasia
ROC: Receiver operating characteristic
AUC: Area under the receiver operating characteristic curve
PSA: Prostate-specific antigen
HGPCa: High-grade prostate cancer
exRNA: Exosomal RNA
exLR: Extracellular long RNA
HCC: Hepatocellular carcinoma
PDAC: Pancreatic ductal adenocarcinoma
IGV: Integrative Genomics Viewer
RT-PCR: Reverse transcription polymerase chain reaction
qPCR: Quantitative polymerase chain reaction
LASSO: Least absolute shrinkage and selection operator.
