# Supplementary material for: Circulating exosomal mRNA profiling identifies novel signatures for the detection of prostate cancer
Source: Mol Cancer. 2021 Mar 30;20:58. doi: 10.1186/s12943-021-01349-z (PMC8008633; doi:10.1186/s12943-021-01349-z)
Supplement: Supplementary file 1 — Additional file 1: Figure S1. Quality control of exosomes isolation. Figure S2. Identificationc the existing forms of circulating emRNAs. Figure S3. Selection of potential diagnostic exosomes mRNA in the LASSO model. Figure S4. Optimized detection strategy for the detection of 13 PCa-associated emRNAs. Figure S5. Scatter plots of emRNA expression validation. Figure S6. Testing of previous reported reference genes. Figure S7. Standard curve generated with real-time quantitative PCR. Figure S8. Established the subtype signatures for the detection of PCa. Figure S9. EmRNAs are derived from PCa and then released into the cell culture medium or circulation by packing into exosomes. Figure S10. The potential biological function of the emRNAs. Table S1. The list of dysregulated transcripts with varied expression between tissue and serum exosomes. Table S2. The list of primers and probes. Table S3. The list of upregulated emRNA in PCa. Table S4. Diagnosis performance of emRNAs. Table S5. Demographics of PCa patients and control participants for QC of exosome isolation. Table S6. Demographics of PCa patients and control participants for RNA-seq of their serum exosome. Table S7. Demographcs of PCa patients and control participants for dysregulated emRNAs validation. Table S8. Demographics of PCa patients and control participants for TaqMan qPCR testing. Table S9. Diagnosis performance of emRNAs in different PSA group. Table S10. Diagnosis performance of emRNAs in different ages. Table S11. Diagnosis performance of emRNAs in differentiating BPH and PCa with GS 6 from PCa with GS ≥7. Identification the existing forms of circulating emRNAs. Optimized detection strategy for the detection of 13 PCa-associated emRNAs. Established the subtype signatures for the detection of PCa. Correlation analysis between emRNAs and the grade of PCa aggressiveness. The source of circulating emRNAs. The potential importance of the emRNAs. Methods. [file 12943_2021_1349_MOESM1_ESM.docx]

**Additional File 1**

**Additional File 1:** **Figure S1**


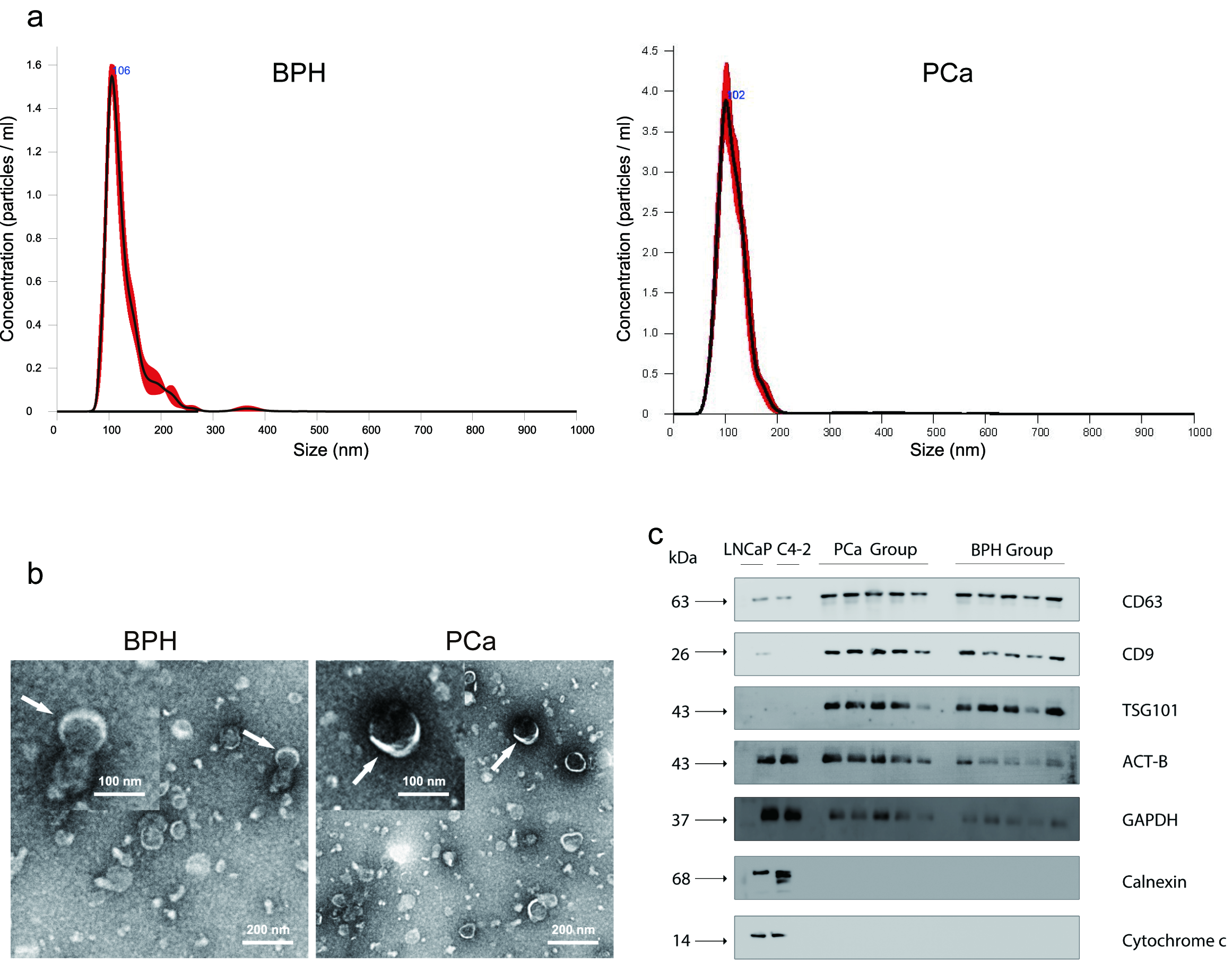


**Additional File 1: Figure S1 Quality control of exosomes isolation.** ***a***, The nano track analysis shows the particle size distribution of exosomes in BPH and PCa patients. ***b***, TEM graphs of exosomes isolation by commercial kit of BPH and PCa patients. ***c***, Western blotting for exosomal markers CD63, CD9, TSG101, ACTB and GAPDH and for non-exosomal markers Calnexin and Cytochrome c.

**Additional File 1:Figure S2**

**Additional File 1:Figure S2 Identificationc the existing forms of circulating emRNAs.** ***a***, Genomics Viewer (IGV) to visualize the reads distribution across the transcriptome of *KLK3*, *AR*, *FOXA1* in exosomes and tissue; ***b***, RT-PCR to amplify the whole transcripts of *KLK3*, *AR* and *FOXA1* in tissues, cell lines, exosomes from serum and exosomes from cell culture medium; ***c***, Different expression level of *KLK3* amplicons identified by qPCR in serum exosomes from PCa and BPH.

**Additional File 1: Figure S3**


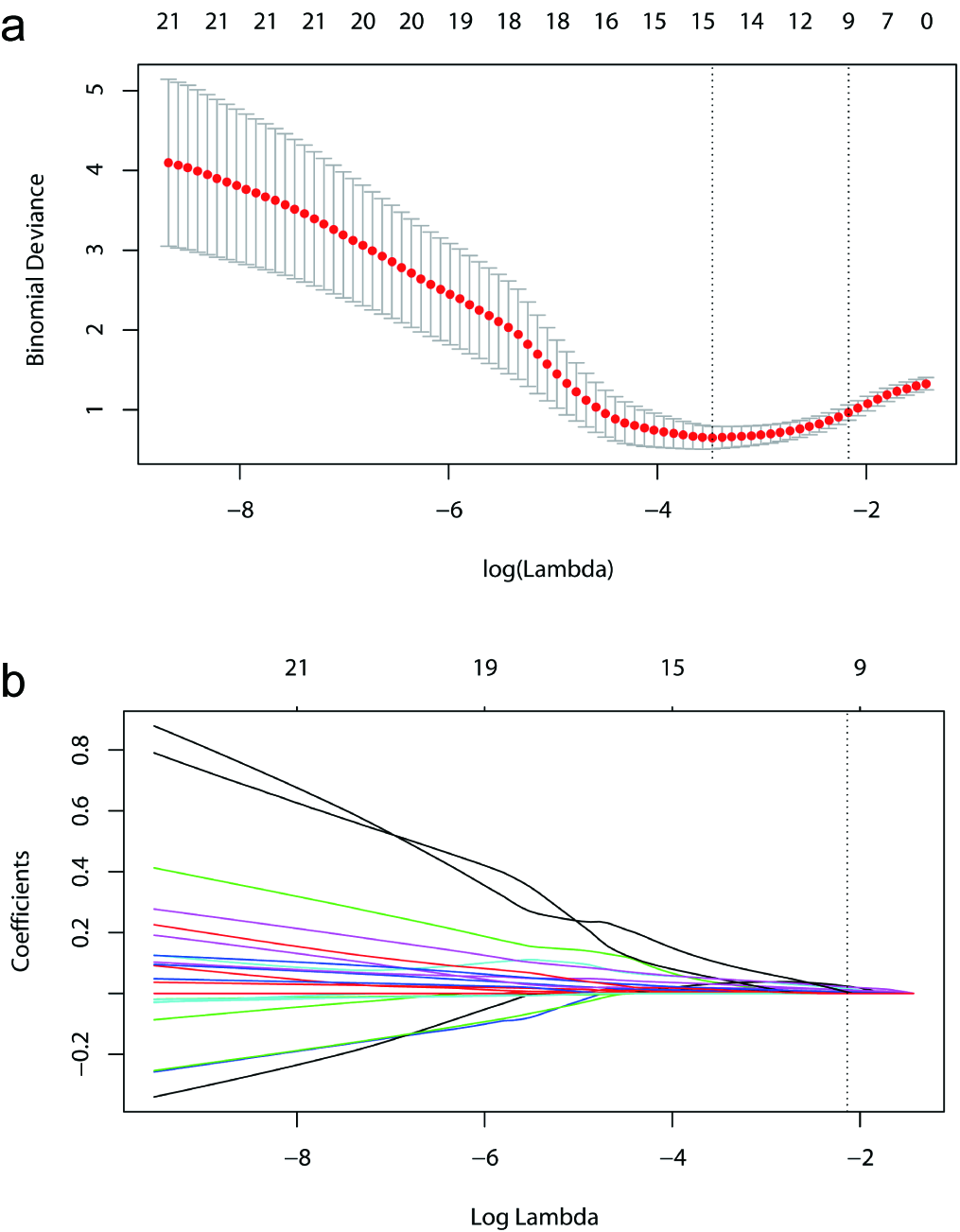


**Additional File 1: Figure S3 Selection of potential diagnostic exosomes mRNA in the LASSO model.** ***a,*** Ten-fold cross-validations for tuning parameter selection in the LASSO model. The solid vertical lines: partial likelihood deviance ± standard error (SE). The dotted vertical lines: optimal values by minimum criteria and 1-SE criteria. We plotted the partial likelihood deviance versus log (λ), where λ is the tuning parameter. We identified a λ of 0.1251647 based on via 1-SE criteria. ***b,*** LASSO coefficient profiles of the 38 biomarkers. A vertical line is drawn at the lambda value chosen by ten-fold cross-validation.

**Additional File 1:Figure S4**

**

**

**Additional File 1:Figure S4 Optimized detection strategy for the detection of 13 PCa-associated emRNAs.** ***a***, work-flow, including identifying emRNA candidates by RNA sequencing, visualizing the reads distribution of emRNAs by IGV, validating the primers by RT-PCR, and designing qPCR probes for emRNA detection. ***b***, Genomics Viewer (IGV) to visualize the reads distribution across the transcriptome of 13 PCa-associated emRNAs (*TXK*, *ATM*, *TOX4*, *MAX*, *STK4*, *GRK5*, *PDGFA*, *RASSF5*, *IL32*, *CDC42*, *FAM228B*, *NCF2* and *SRSF2*). ***c***, Validated the multiple primers for different regions in the exons of each emRNA by reverse transcription polymerase chain reaction (RT-PCR).

**Additional File 1: Figure S5**


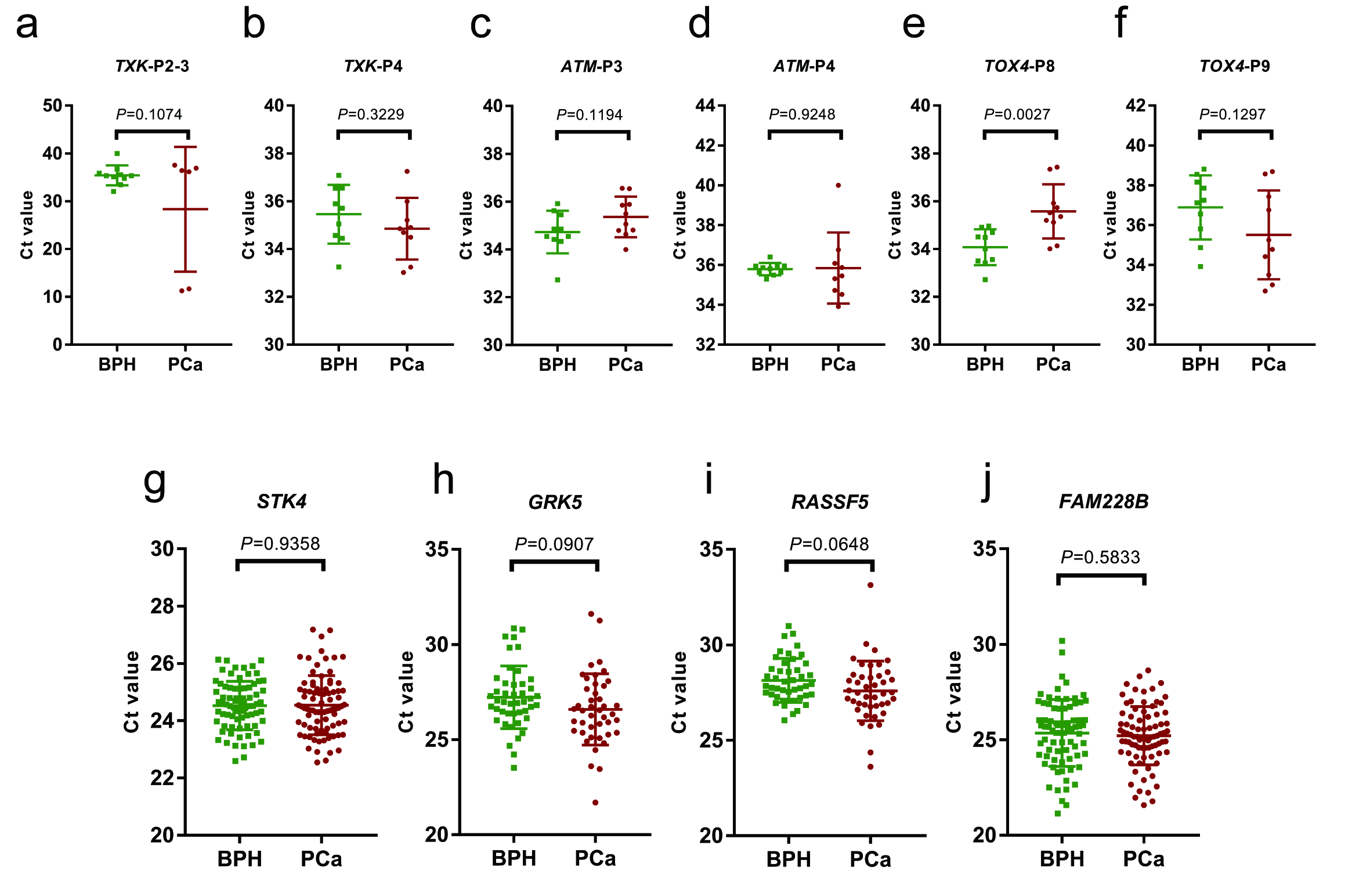


**Additional File 1: Figure S5 Scatter plots of emRNA expression validation. *a-f*,** TaqMan qPCR validation of the expression of 6 PCa-associated emRNAs in 10 PCa patients and 10 controls (n=10, Student’s t tset). ***g-I,*** TaqMan qPCR validation of 4 emRNAs identified by emRNA-sequencing in an independent cohort of 84 PCa patients and 76 controls.

**Additional File 1: Figure S6**

**
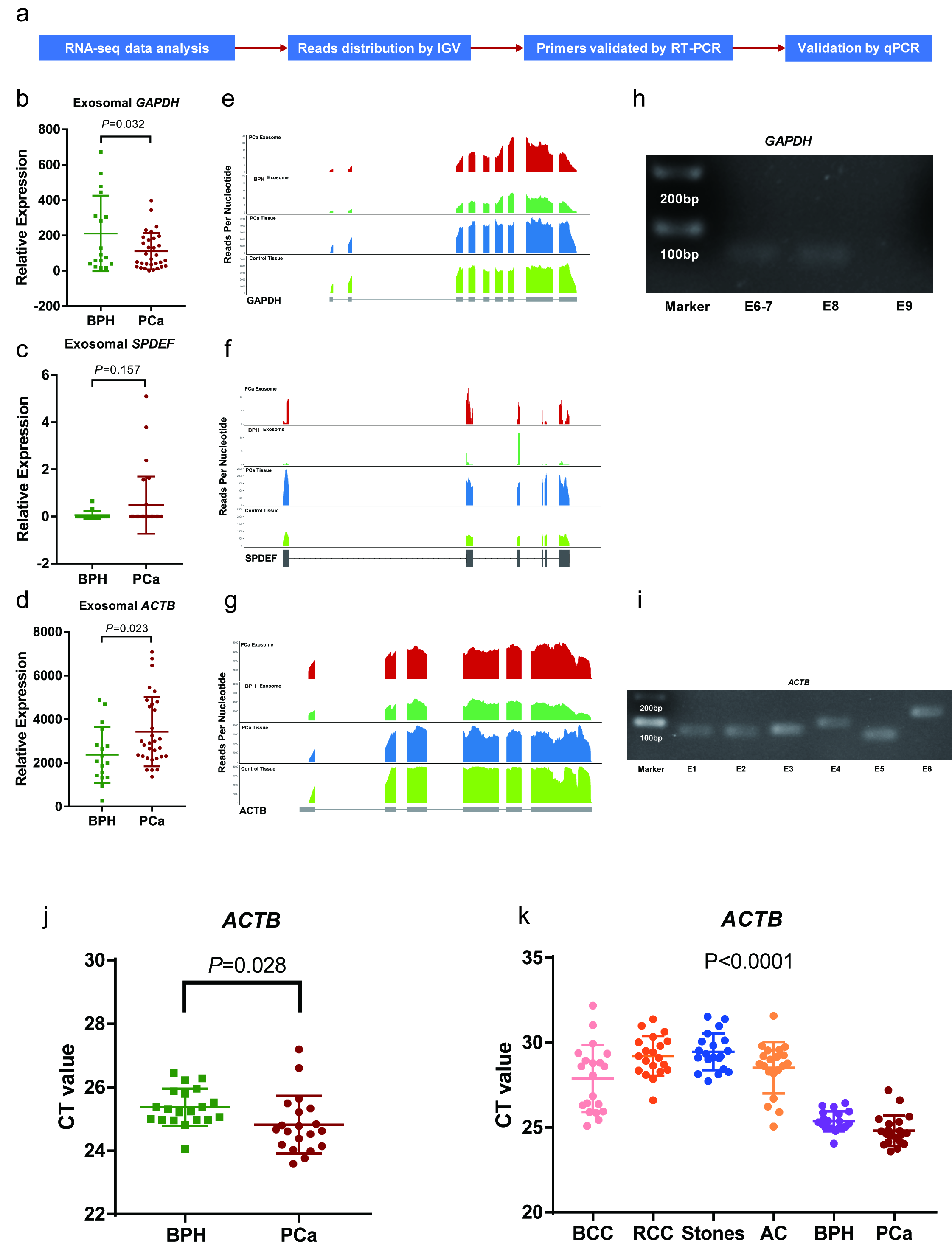
**

**Additional File 1: Figure S6 Testing of previous reported reference genes.** ***a***, Workflow summary. RNA-seq demonstrates the expression levels of *GAPDH* (***b***), SPDEF (***c***), and *ACTB* (***d***) between PCa and control. Reads distribution identified by IGV (***e*-*g***). ***h***, *GAPDH* was not detected in circulating exosomes. ***i****,* *ACTB* was detected in exosomes. The results of RT-qPCR validation showed the *ACTB* expression level in PCa (***j***) (control=20, PCa=20, Student’s t tset) and other diseases (***k***) including breast cancer (BCC), renal cell carcinoma (RCC, urinary stones (stones), AC, benign prostatic hyperplasia (BPH) and PCa (n=20, one-way ANOVA).

**Additional File 1: Figure S7**


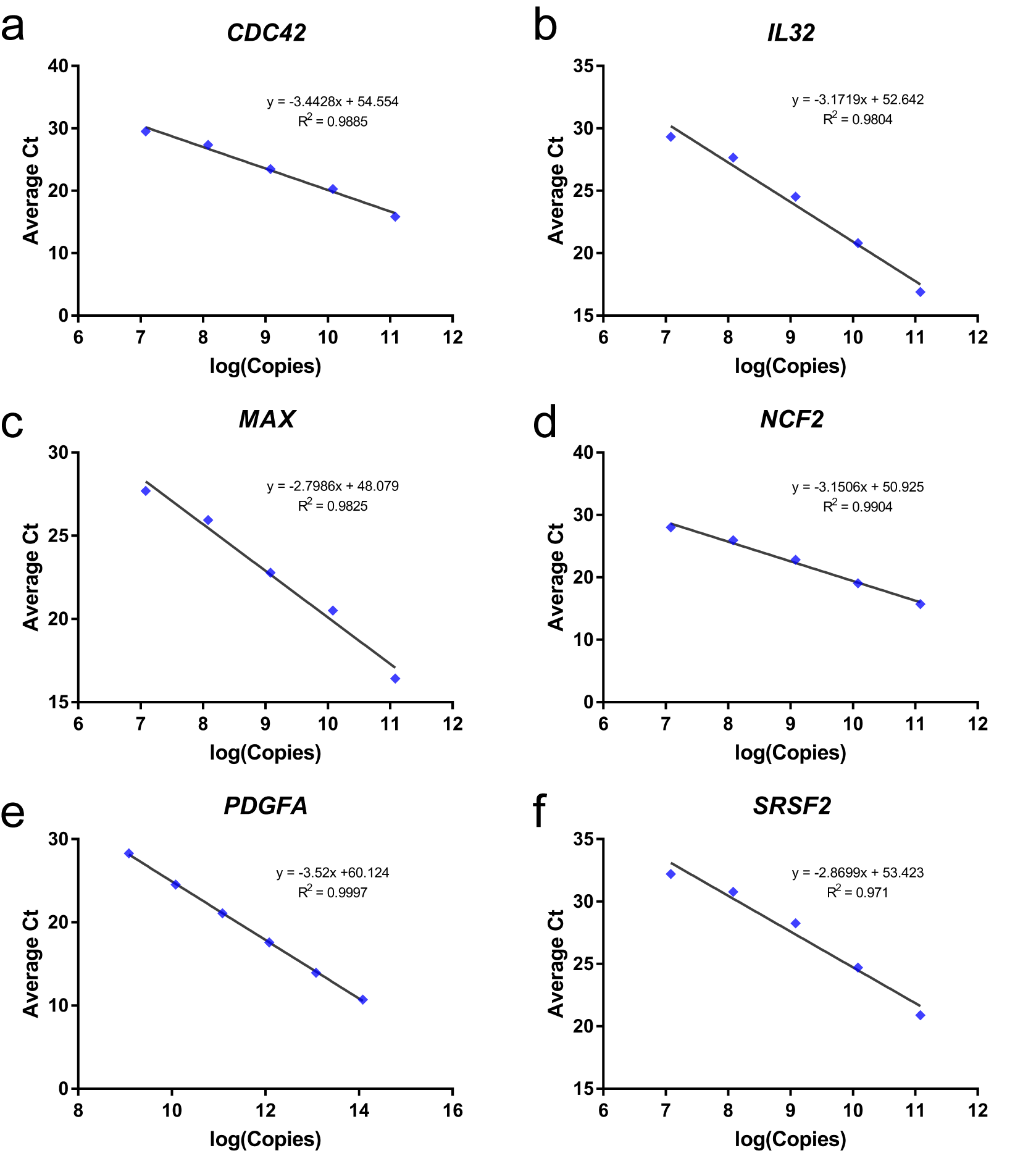


**Additional File 1: Figure S7 Standard curve generated with real-time quantitative PCR** by testing synthesized transcripts at different copy number concentration gradients *CDC42* (***a***), *IL32* (***b***), *MAX* (***c***), *NCF2* (***d***), *PDGFA* (***e***) and *SRSF2* (***f***) in serum exosomes.

**Additional File 1: Figure S8**


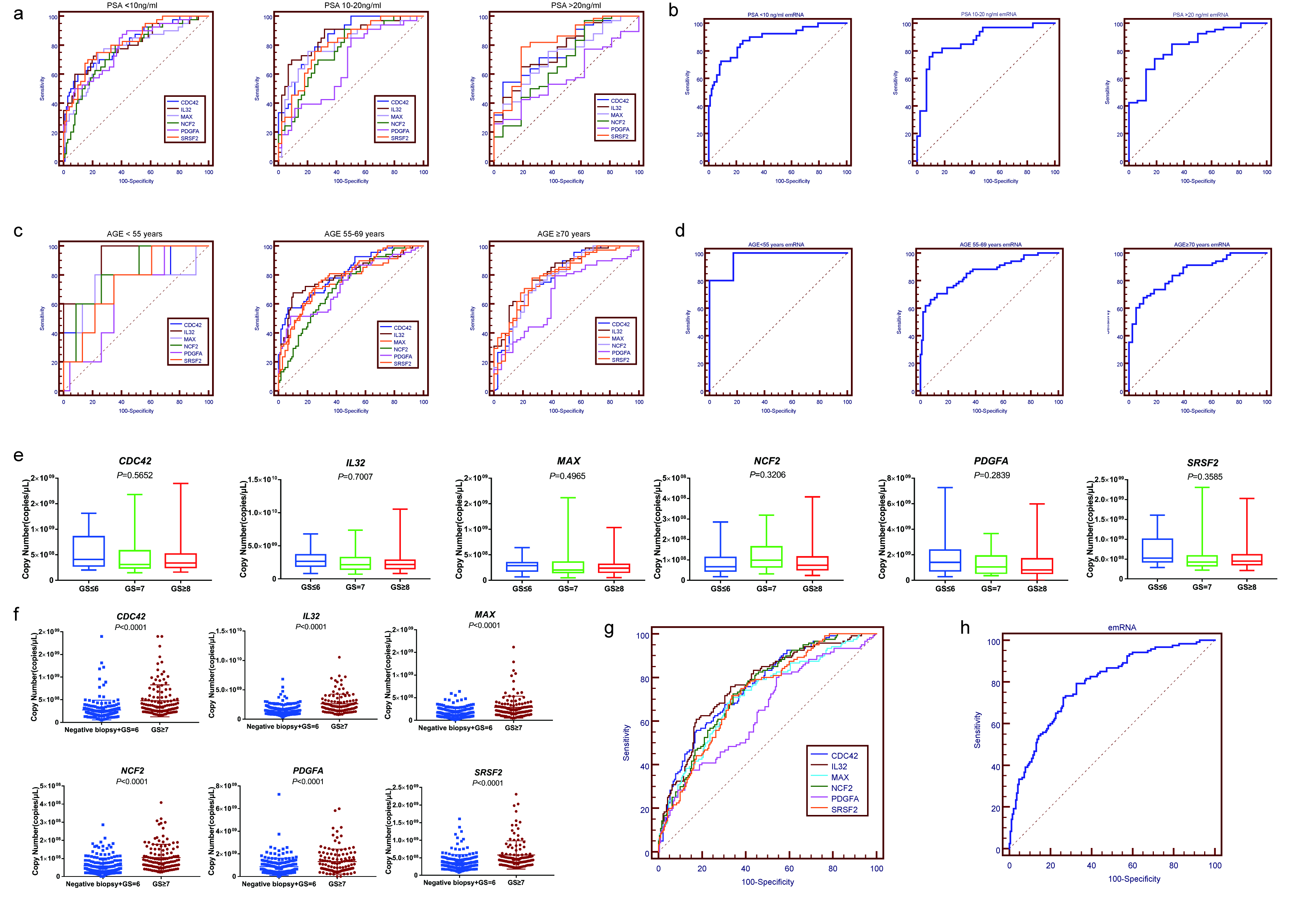


**Additional File 1:Figure S8 Established the subtype signatures for the detection of PCa. *a*,** ROC analysis shows the diagnostic performance of *CDC42*, *IL32*, *MAX*, *NCF2*, *PDGFA* and *SRSF2* in PSA-based groups, PSA <10ng/ml, PSA 10-20ng/ml and PSA >20ng/ml. The detailed information was summarized in **Additional File 1: Table S9**. ***b***, ROC analysis shows the diagnostic performance of PSA-based subgroups (AUC of 0.884 in PSA <10ng/ml group, 0.869 in PSA 10-20ng/ml, and 0.835 in PSA >20ng/ml group). The detailed information was summarized in **Additional File 1:Table S9**. ***c***, ROC analysis shows the diagnostic performance of CDC42, IL32, MAX, NCF2, and SRSF2 in age-based groups, <55 years, 55-69 years and >70 years. The detailed information was summarized in **Additional File 1:Table S10**. ***d***, ROC analysis shows the diagnostic performance of PSA-based subgroups (AUC of 0.965 in <55 years group, 0.855 in 55-69 years group, and 0.855 in >70 years group). The detailed information was summarized in **Additional File 1:Table S10**. ***e***, Correlation analysis between 6 emRNAs and the grade of PCa aggressiveness. ***f***, Scatter plots show the expression levels of circulating emRNAs, including *CDC42*, *IL32*, *MAX*, *NCF2*, *PDGFA* and *SRSF2*, between HGPCa patients and BPH plus LGPCa. ***g***, ROC analysis shows the diagnostic performance of *CDC42*, *IL32*, *MAX*, *NCF2*, *PDGFA* and *SRSF2* for HGPCa diagnosis. The detailed information was summarized in **Additional File 1:Table S11**. ***h***, ROC analysis shows the diagnostic performance of emRNA-based diagnostic model for HGPCa diagnosis.

**Additional File 1:Figure S9**

**
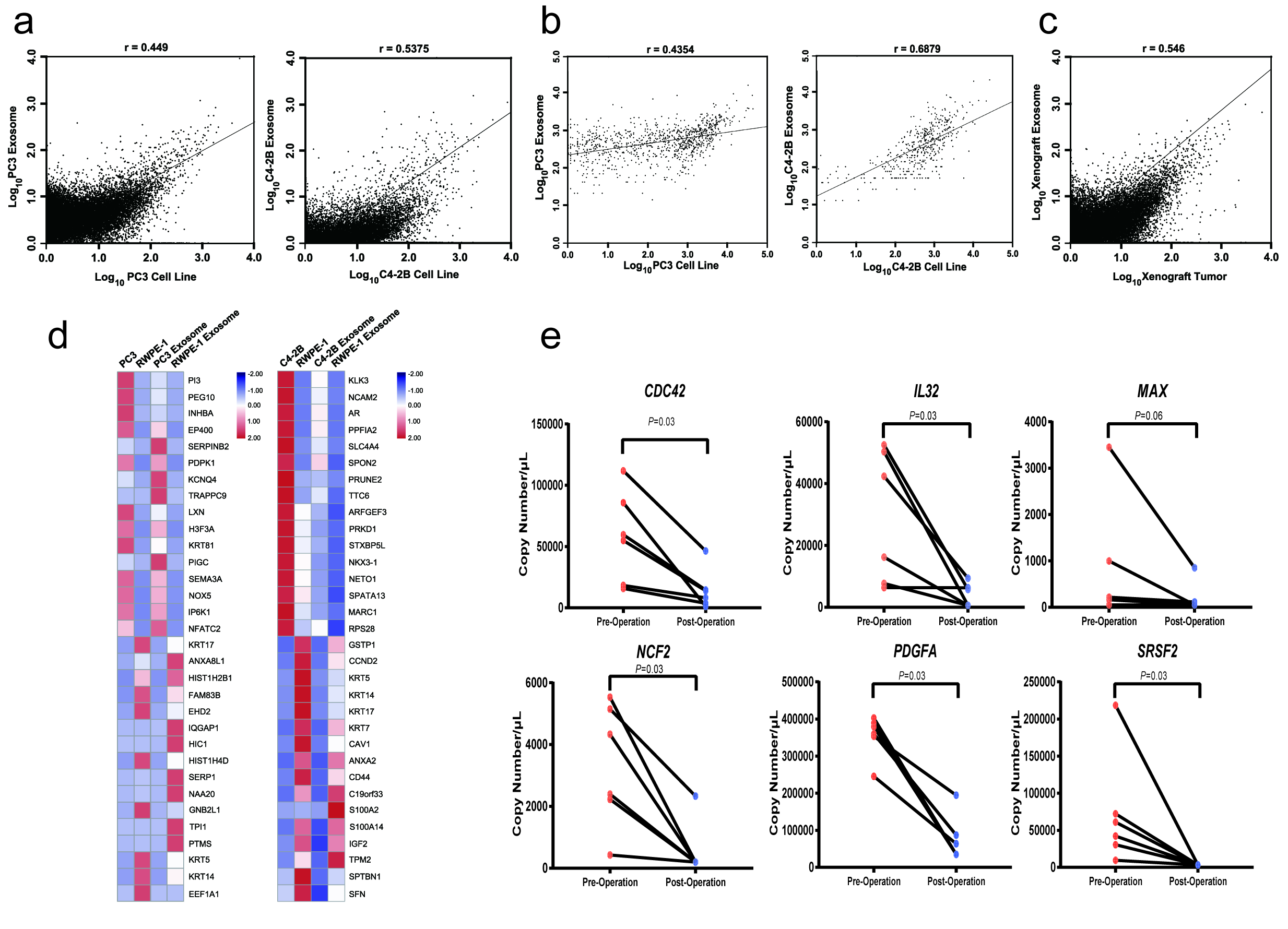
**

**Additional File 1: Figure S9.** **EmRNAs are derived from PCa and then released into the cell culture medium or circulation by packing into exosomes.** Scatter plot illustrating the correlation between all mRNAs (***a***) and oncogene mRNAs (***b***) in the cell lines vs. exosomes from culture supernatants of the PC3 and C4-2B cell lines. ***c***, Scatter plot illustrating the correlation between all mRNAs in exosomes from peripheral blood of the PC3 mouse xenograft and the tumor from the xenograft. ***d***, HeatMap shows that the dysregulated mRNAs in PCa and C4-2B cells compared to those in control RWPE-1 cells demonstrate the same trend in exosomes from PCa cell culture medium compared to that from control cell culture medium. ***e***, The expression levels of 6 representative PCa-associated circulating emRNAs, *CDC42*, *IL32*, *MAX*, *NCF2*, *PDGFA* and *SRSF2*, in PCa patients (n=6) decreased significantly one month after radical prostatectomy.

**Additional File 1:Figure S10**


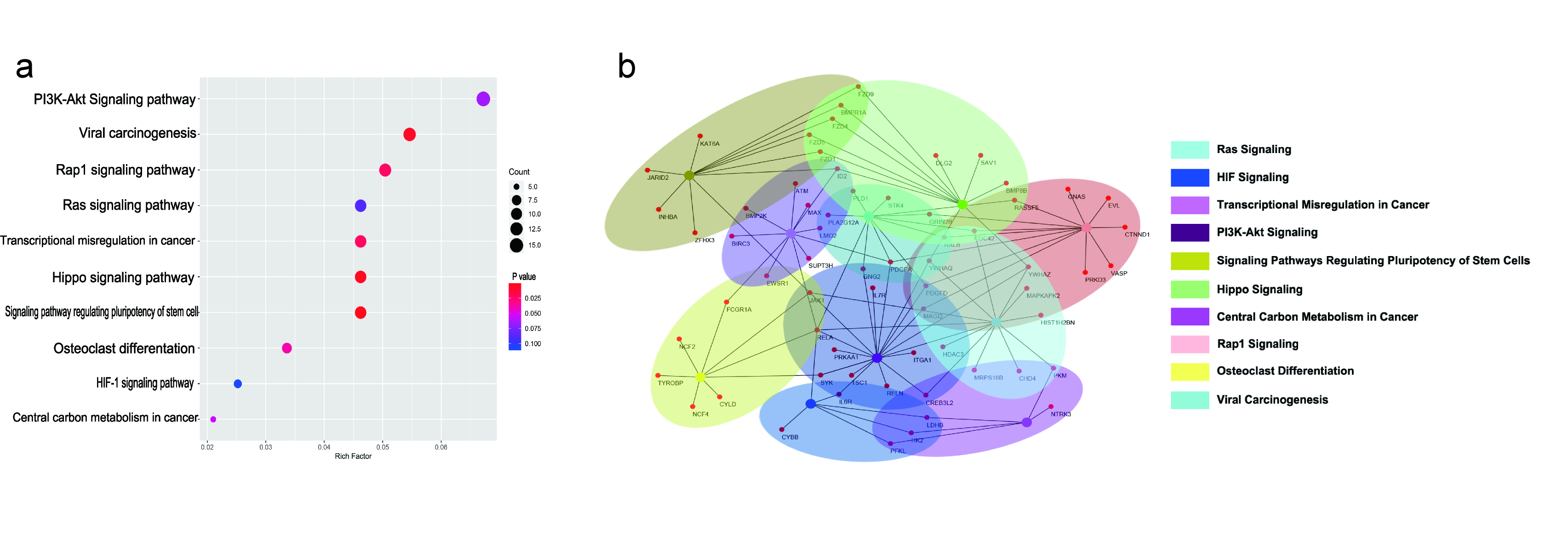


**Additional File 1:Figure S10 The potential biological function of the emRNAs** ***a,***KEGG pathway enrichment analysis. ***b,*** Molecular network of the relevant pathways.

**Additional File 1: Table S1** The list of dysregulated transcripts with varied expression between tissue and serum exosomes

| Both up-regulated in exRNA and tissue | | | |
| --- | --- | --- | --- |
| Transcript_id | gene_name | exRNA | Tissue |
| ENST00000216129 | TTLL12 | 1.635141143 | 1.166280711 |
| ENST00000269391 | RNF157 | 2.7882788 | 2.212724599 |
| ENST00000277141 | [TUT7](https://www.ncbi.nlm.nih.gov/gene/79670) | 1.077790612 | 1.163684802 |
| ENST00000338981 | USP9Y | 1.556809213 | 1.132142445 |
| Up-regulated in exRNA and down-regulated in tissue | | | |
| Transcript_id | gene_name | exRNA | Tissue |
| ENST00000267205 | RHOF | 1.052517778 | -1.219531315 |
| ENST00000350669 | LDHB | 1.305716534 | -1.401492393 |
| ENST00000392870 | GRK5 | 2.244614749 | -1.271072301 |
| ENST00000396290 | ID2 | 1.785858121 | -1.223677114 |
| ENST00000408965 | CEBPD | 1.433447775 | -1.301925339 |
| ENST00000586839 | AES | 2.180625364 | -1.082837677 |
| ENST00000617275 | PLEKHA2 | 1.270434001 | -3.806232041 |
| Down-regulated in exRNA and up-regulated in tissue | | | |
| Transcript_id | gene_name | exRNA | Tissue |
| ENST00000233969 | SLC9A2 | -2.479086184 | 1.491224162 |
| ENST00000244623 | OR2B6 | -3.226877016 | 2.456555645 |
| ENST00000265000 | GALNT7 | -1.695792758 | 1.775157599 |
| ENST00000278379 | SLC1A2 | -2.376204908 | 1.026740237 |
| ENST00000296043 | SHROOM3 | -2.902811143 | 1.015938073 |
| ENST00000304916 | FAM84B | -1.220153564 | 1.324165288 |
| ENST00000315251 | CHDH | -2.432233923 | 1.170199562 |
| ENST00000410061 | RAB6C | -3.378200749 | 1.672282117 |
| ENST00000431473 | RIMKLA | -3.313065917 | 1.419913487 |
| ENST00000491143 | ONECUT2 | -2.656121378 | 2.209813511 |
| ENST00000521891 | ZFHX4 | -2.365149751 | 1.981093652 |
| ENST00000606613 | HIST1H2BN | -3.982605097 | 1.570929595 |
| ENST00000612452 | AR | -1.521371736 | 1.205490328 |
| Both down-regulated in exRNAand tissue | | | |
| Transcript_id | gene_name | exRNA | Tissue |
| ENST00000217289 | FERMT1 | -3.229447206 | -1.343905679 |
| ENST00000219454 | WFDC1 | -3.059471032 | -1.688050535 |
| ENST00000250111 | ATP1B2 | -1.796730594 | -1.177373655 |
| ENST00000258104 | DYSF | -1.300398967 | -2.046759968 |
| ENST00000262426 | FOXF1 | -5.130510697 | -1.746565459 |
| ENST00000265362 | SEMA3A | -2.803345333 | -1.414396303 |
| ENST00000280481 | FREM2 | -4.079453986 | -1.300278633 |
| ENST00000282588 | ITGA1 | -2.199834072 | -1.111369687 |
| ENST00000296795 | TLR3 | -3.239395616 | -1.092358786 |
| ENST00000300571 | GPRC5B | -1.872160499 | -1.215493848 |
| ENST00000359450 | TIAF1 | -2.004461438 | -1.000819883 |
| ENST00000368081 | ATP1A4 | -2.899741672 | -3.081230655 |
| ENST00000369780 | NEURL1 | -3.524202159 | -1.162899328 |
| ENST00000376020 | SHROOM4 | -2.749926721 | -1.355765003 |
| ENST00000380872 | AKR1C1 | -3.154623515 | -1.07412963 |
| ENST00000381280 | SMOC1 | -5.534536435 | -2.354156189 |
| ENST00000394480 | NTRK3 | -2.902583962 | -1.663387004 |
| ENST00000442544 | DCC | -3.175228926 | -1.548654283 |
| ENST00000490531 | NACAD | -2.280006798 | -1.802605615 |
| ENST00000506151 | USP17L20 | -4.261472851 | -1.806133567 |
| ENST00000515384 | AMER2 | -2.112528984 | -2.294618452 |
| ENST00000527524 | GRIK4 | -3.075722862 | -1.448332133 |
| ENST00000548058 | ACSS3 | -3.853105974 | -1.267727916 |
| ENST00000581347 | TMEM200C | -3.490516534 | -1.950622873 |
| ENST00000606738 | TRABD2B | -4.932487666 | -1.215090916 |
| Up-regulated and down-regulated in exRNA, but no significant difference compared with tissue | | | |
| Transcript_id | gene_name | exRNA | Tissue |
| ENST00000219837 | KNOP1 | -1.575574432 | 0.288890637 |
| ENST00000223023 | WASL | -1.218783599 | 0.228227541 |
| ENST00000228515 | CSRNP2 | -1.458061952 | -0.455596828 |
| ENST00000231572 | RARS | 1.224377164 | 0.475385029 |
| ENST00000234160 | GORASP2 | 1.705868766 | 0.52419948 |
| ENST00000237596 | PKD2 | -2.146742147 | -0.685687148 |
| ENST00000243501 | PLA2G12A | 1.204154291 | 0.725290694 |
| ENST00000245932 | VASP | 1.002698899 | -0.483091639 |
| ENST00000262126 | ANKRD12 | 1.026627872 | -0.768497009 |
| ENST00000264065 | DNAJC10 | -1.430431149 | 0.832068368 |
| ENST00000269142 | TAF4B | -3.465970129 | 0.611418722 |
| ENST00000271764 | EIF2D | 1.766476315 | 0.2989512 |
| ENST00000280979 | AKAP6 | -3.260050921 | -0.875296832 |
| ENST00000285930 | AKR1B1 | 1.639685642 | -0.607004549 |
| ENST00000290573 | HK2 | 1.694035979 | 0.576697641 |
| ENST00000296318 | IL17RD | -3.59803512 | -0.887179624 |
| ENST00000296666 | PRRC1 | 1.466943928 | 0.507029391 |
| ENST00000297290 | BRI3 | 1.595173292 | 0.577493685 |
| ENST00000303391 | MECP2 | 1.527983695 | -0.817699578 |
| ENST00000306065 | ANKRD27 | 1.1748664 | -0.265402694 |
| ENST00000307221 | DNAJB7 | -3.250296297 | 0.158984891 |
| ENST00000311672 | UQCRH | 1.269580496 | 0.657179332 |
| ENST00000317005 | TIGD2 | -4.329557771 | 0.63155772 |
| ENST00000321301 | TOMM5 | 2.201015689 | 0.571813259 |
| ENST00000322054 | EHD3 | 1.926335343 | -0.724002613 |
| ENST00000323061 | NAP1L5 | -1.826484377 | -0.406507482 |
| ENST00000326840 | DCBLD2 | -1.743502348 | -0.6354742 |
| ENST00000338728 | DCBLD1 | -5.187512018 | 0.447641972 |
| ENST00000359520 | TECPR2 | 1.521440088 | -0.530373488 |
| ENST00000360187 | ZNF605 | -2.26634133 | 0.387885577 |
| ENST00000361203 | DST | -1.493476905 | -0.81151508 |
| ENST00000366956 | PTPN14 | -2.067071813 | -0.744758286 |
| ENST00000369103 | RGS10 | 1.245755421 | 0.749225916 |
| ENST00000369209 | HSPA12A | -4.987062586 | -0.981002743 |
| ENST00000370192 | DPYD | 1.250831968 | -0.629065243 |
| ENST00000371717 | PMPCA | 2.744420383 | 0.264888854 |
| ENST00000372409 | PCIF1 | 1.271939693 | -0.62935182 |
| ENST00000373191 | AGO3 | -1.065579588 | -0.517594964 |
| ENST00000374980 | EIF2S2 | 1.529046113 | 0.39755221 |
| ENST00000375151 | TSR2 | 1.655960003 | -0.307745383 |
| ENST00000377093 | KIF1B | -1.663368033 | -0.463539064 |
| ENST00000379066 | PRKD3 | 1.048956907 | -0.444335024 |
| ENST00000379359 | RGCC | 1.110039504 | -0.785375477 |
| ENST00000380916 | ZDHHC21 | -1.255459371 | 0.336644506 |
| ENST00000381989 | PARP4 | 1.106257361 | 0.230698287 |
| ENST00000395310 | SEC31A | 1.371829775 | -0.745875275 |
| ENST00000405409 | RBFOX2 | -1.551047279 | -0.927668491 |
| ENST00000408939 | TTC30B | -2.053682874 | 0.489276633 |
| ENST00000428216 | MAVS | -1.50227335 | 0.125118323 |
| ENST00000433060 | AMOTL1 | -1.243156244 | -0.564168942 |
| ENST00000442138 | PRRC1 | -1.384160409 | 0.501175574 |
| ENST00000442263 | INTS6 | -2.386779303 | -0.555009753 |
| ENST00000470557 | PTRH2 | -1.906665376 | 0.244578537 |
| ENST00000479870 | TCAF1 | 1.555758259 | 0.785941215 |
| ENST00000500893 | ZCCHC3 | -1.013670112 | -0.495536299 |
| ENST00000520547 | EIF5AL1 | -2.383407021 | 0.599413965 |
| ENST00000531380 | FZD4 | -1.531759214 | 0.783070173 |
| ENST00000537226 | ZNF891 | -2.948431756 | 0.035469485 |
| ENST00000543111 | FAM186A | -5.11846422 | 0.412562642 |
| ENST00000548580 | MYL6 | 3.2169168 | -0.716197056 |
| ENST00000550735 | MAPKAPK5 | -1.212818785 | 0.190071049 |
| ENST00000556766 | GNG2 | 1.400891018 | -0.929628486 |
| Up-regulated and down-regulated in tissue, but no significant difference compared with exRNA | | | |
| Transcript_id | gene_name | exRNA | Tissue |
| ENST00000202773 | RPL6 | 0.997617084 | 1.260257788 |
| ENST00000317968 | PDLIM5 | 0.903807695 | 2.57379277 |
| ENST00000286713 | STOM | 0.791096161 | -1.268579531 |
| ENST00000380698 | SERPINB9 | 0.801871708 | -1.076656584 |
| ENST00000380739 | SERPINB1 | 0.752812395 | -1.153062055 |
| ENST00000399075 | C4orf3 | 0.883936336 | -1.133052747 |
| ENST00000560626 | PEAK1 | -0.98657343 | -1.867039604 |

**Additional File 1: Table S2** The list of primers and probes

| NO; | Gene name | Primer sequence | Product length (bp) | Probe sequence |
| --- | --- | --- | --- | --- |
| 1 | *ATM*-E2-F | AAATTGTGAACCATGAGTC | 83 | / |
|  | *ATM*-E2-R | TTTCGTTCTGTAGCTCTATC |  |  |
| 2 | *ATM*-E3-F | TTAAGCGCCTGATTCG | 91 | vic-ATCCTGAAACAATTAAACATCT-mgb |
|  | *ATM*-E3-R | ACAGCATCCCAATTCAAA |  |  |
| 3 | *ATM*-E4-F | AATGTCTGAGAATAGCAAAAC | 92 | vic-CCTGCATCTTTTTCTGCCTGGA-mgb |
|  | *ATM*-E4-R | GTATTTGACCAAACTACTGATT |  |  |
| 4 | *ATM*-E16-F | TTGACCGTGGAGAAGTAGA | 101 | / |
|  | *ATM*-E16-R | ATCAGGGTAATCGTTAAATAGA |  |  |
| 5 | *GRK5*-E2-F | CAAAGGGAAAAGCAAGAAGT | 80 | vic-AAAGAAATCCTGAAGTTCCCT-mgb |
|  | *GRK5*-E2-R | CTATGGTCCTTCGGAGGTC |  |  |
| 6 | *GRK5*-E3-F | TTTATGTGACAAGCAGCCAATC | 95 | vic-CTGCTTTTCCGGCAGTTTTGT-mgb |
|  | *GRK5*-E3-R | CGGAGTCCAGGAACTGAATGT |  |  |
| 7 | *GRK5*-E4-F | AGAATATGAAGTTACTCCAGATG | 72 |  |
|  | *GRK5*-E4-R | GGGGTGAGGTACTTGGTC |  |  |
| 8 | *IL32*-E2-3-F | GCCTTGGCTCCTTGAACTTTT | 82 | / |
|  | *IL32*-E2-3-R | CATTCGGGCCTTCAGCTTCT |  |  |
| 9 | *IL32*-E5-6-F | GTGATGTCGAGCCTGGCA | 86 |  |
|  | *IL32*-E5-6-R | GGGTGCTGCTCCTCATAATAAG |  |  |
| 10 | *IL32*-E7-F | CTGGGTGAAGGAGAAGGTGG | 109 | fam-CCCTGGTCCATGCAGTGCAGGCC-mgb |
|  | *IL32*-E7-R | CTGGAAAGAGGACATGAAGAGC |  |  |
| 11 | *MAX*-E1-F | CTCGGCTTGTTGTTGTCGG | 107 | fam-CCCAGGGAGCGGCCACTGCAGC-mgb |
|  | *MAX*-E1-R | CATCGTTATCGCTCATTTCCTAC |  |  |
| 12 | *MAX*-E3-F | CAAATCCTAGACAAAGCCACA | 111 |  |
|  | *MAX*-E3-R | TTGCTGCTCCAGAAGAGC |  |  |
| 13 | *MAX*-E6-F | TGAGAAGATGACCGGTTTGGA | 112 | fam-TAATCAGAATGAACCCTCCTCCT-mgb |
|  | *MAX*-E6-R | GATCTGGTCTTTCAATAGGAG |  |  |
| 14 | *PDGFA*-E1-F | AACGCACCGAGGAAGAAGC | 119 | / |
|  | *PDGFA*-E1-R | CAGGAGGAGGAGAAACAGGGA |  |  |
| 15 | *PDGFA*-E4-F | GACGGTCATTTACGAGATTCCTC | 92 |  |
|  | *PDGFA*-E4-R | TGCAGCGTTTCACCTCCAC |  |  |
| 16 | *PDGFA*-E5-F | CCAAGGTGGAATACGTCAGGA | 99 |  |
|  | *PDGFA*-E5-R | GATTCAGGCTTGTGGTCGC |  |  |
| 17 | *PDGFA*-E6-F | TGTGCGGTCTTTGTTCTCCTC | 111 | fam-CACATTAAACAAATGTGCAC-mgb |
|  | *PDGFA*-E6-R | CGAGTGCTACAATACTTGCTTTGA |  |  |
| 18 | *RASSF5*-E2-F | TCTGTAAACCTGTGGAGGAGAC | 101 | / |
|  | *RASSF5*-E2-R | CATGCCCAGGCAGTTCTTC |  |  |
| 19 | *RASSF5*-E3-F | ACCACGGACAAGCGGACAT | 106 | vic-CCTTCTACCTGCCCCTAGAT-mgb |
|  | *RASSF5*-E3-R | TCTTGAGCAGCCCCTGGAT |  |  |
| 20 | *RASSF5*-E5-F | AGGTGCTGCCCAAGGAGTT | 92 | vic-CTCCCCTCTCTGTAATCAAGG-mgb |
|  | *RASSF5*-E5-R | TCTGGCCCATGCTTCAAAA |  |  |
| 21 | *STK4*-E7-F | AATCCTCCTCCCACATTCCG | 91 |  |
|  | *STK4*-E7- R | TCTGCTCAGGGCTCTTTACAAG |  |  |
| 22 | *STK4*-E8-F | CCCATTTGTCAGGAGTGCC | 100 |  |
|  | *STK4*-E8- R | TTCCCGCTGCTGGGATT |  |  |
| 23 | *STK4*-E9-F | GATGGTTCGAGCAGTGGGTG | 102 | fam-CAATCATAGTATTGGCTCCATC- mgb |
|  | *STK4*-E9- R | GATGGCAACGTGTCATCGTG |  |  |
| 24 | *STK4*-E11-F | AGGGAACCTTGCTAACAGAAAC | 109 | / |
|  | *STK4*-E11- R | AAGGACTCGCTGAAACTGAATG |  |  |
| 25 | *TOX4*-E1-F | CAATTTGGGAGCTTCG | 63 | / |
|  | *TOX4*-E1-R | CACAGGTAACCCTCAGGT |  |  |
| 26 | *TOX4*-E8-F | GTCTCAAGGAGGGATGGTTACT | 113 | fam-CCCAGCCACAGTGGTGACCTCC-mgb |
|  | *TOX4*-E8-R | CAATCTGGGCTTGTTGACTGG |  |  |
| 27 | *TOX4*-E9-F | TGGGTCTCCTGTGGCACTCT | 97 | fam-CCAGCCTCGATGTGTGAGGTC-mgb |
|  | *TOX4*-E9-R | GCAGTATTCATTGTCCCAGTCCTT |  |  |
| 28 | *TOX4*-E10-F | TTCAACCATAAGCGGTAATAGCA | 72 | / |
|  | *TOX4*-E10-R | CAACAAGTTTCTTCAACCCCAC |  |  |
| 29 | *TXK*-E2-3-F | AACACCATCCAGTCGGTTTTC | 89 | vic-ACTGAACAGCAACAGCAGCAA- mgb |
|  | *TXK*-E2-3-R | TCATCTGTGCTCAGGCTTATCTG |  |  |
| 30 | *TXK*-E4-F | TGTGCAGCCGTCAAAACG | 100 | vic-CCACTGCCTCCCCTCCCAC - mgb |
|  | *TXK*-E4-R | TCTGGGCAGAAAATCATAAAGTG |  |  |
| 31 | *TXK*-E14-F | GAGTTTTAATGTGGGAAGTTT | 114 | fam-CCAGCCTCGATGTGTGAGGTC- mgb |
|  | *TXK*-E14-R | CCAGGTGAGGGCGATA |  |  |
| 32 | *TXK*-E15-F | TGAAGTCTGACAACAGGAGCCC | 119 | / |
|  | *TXK*-E15-R | GTCGCAACTACTGACAATCAACAA |  |  |
| 33 | *GAPDH*-E67-F | AAGTATGACAACAGCCTCAAGATCA | 110 | / |
|  | *GAPDH*-E67-R | ATGAGTCCTTCCACGATACCAAA |  |  |
| 34 | *GAPDH*-E8-F | ATCAAGAAGGTGGTGAAGCAGG | 150 | / |
|  | *GAPDH*-E8-R | AAAGTGGTCGTTGAGGGCAAT |  |  |
| 35 | *GAPDH*-E9-F | AAGGGGTCTACATGGCAACTGT | 152 | / |
|  | *GAPDH*-E9-R | ACATGGCCTCCAAGGAGTAAGA |  |  |
| 36 | *ACTB*-E1-F | GTGCGCCGTTCCGAAAGTT | 70 | fam-CCGCCGCGGCCGCTCGAGCCATAA-mgb |
|  | *ACTB*-E1-R | CGTCGCGCCGCTGGGTTTTATAGGG |  |  |
| 37 | *ACTB*-E2-F | GCTCCGGCATGTGCAAGG | 70 | fam-CTTCGCGGGCGACGATGCCCC-mgb |
|  | *ACTB*-E2-R | CCCACGATGGAGGGGAAGAC |  |  |
| 38 | *ACTB*-E3-F | TGATGGTGGGCATGGGTC | 82 | fam-CTCGTCGCCCACATAGGAATCCTT-mgb |
|  | *ACTB*-E3-R | GGGTACTTCAGGGTGAGGAT |  |  |
| 39 | *ACTB*-E4-F | GGAAATCGTGCGTGACATT | 113 | fam-CTGTGCTACGTCGCCCTGGACTTC-mgb |
|  | *ACTB*-E4-R | CAGGCAGCTCGTAGCTCTT |  |  |
| 40 | *ACTB*-E5-F | GTGGACATCCGCAAAGAC | 65 | fam-CTGTACGCCAACACAGTGCT-mgb |
|  | *ACTB*-E5-R | CCAGGGTACATGGTGGTG |  |  |
| 41 | *ACTB*-E6-F | TGCGTTACACCCTTTCTT | 150 | fam-CAAAACCTAACTTGCGCAGAAAAC-mgb |
|  | *ACTB*-E6-R | CTGTCACCTTCACCGTTC |  |  |
| 42 | *SPDEF*-E2-F | TTGACAGCCAAGCCCCAG | 106 |  |
|  | *SPDEF* -E2-R | AGCACTTCGCCCACCAC |  |  |
| 43 | *SPDEF* -E5-F | CCGACAGCGAGGTGGA | 115 |  |
|  | *SPDEF* -E5-R | TTGTTGAGCCACCTAATGAAG |  |  |
| 44 | *SPDEF* -E6-F | AACTACGACAAGCTGAGCCG | 76 |  |
|  | *SPDEF* -E6-R | GCTGGGAGATGTCTGGCTTC |  |  |
| 45 | *SPDEF* -E34-F | CCACCTGGACATCTGGAAG | 61 |  |
|  | *SPDEF* -E34-R | AATCGCCCCAGGTGAAGT |  |  |
| 46 | *CDC42*-E3-F | AGCAATGCAGACAATTAAG | 105 |  |
|  | *CDC42*-E3-R | GGTACATATTCCGATGGAA |  |  |
| 47 | *CDC42*-E6-F | ACTCCTTTCTTGCTTGTTGGG | 90 | vic-CAAGTTTCTCAATAGTAGA-mgb |
|  | *CDC42*-E6-R | GATAGGCTTCTGTTTGTTCTTGG |  |  |
| 48 | *CDC42*-E7-F | GAGCCTCCAGAACCGAAGAA |  |  |
|  | *CDC42*-E7-R | GTATGATGCCGACACCAGCT |  |  |
| 49 | *FAM228B*-E4-F | TATAAAAGATGGGTTGACTGT | 100 | fam-CAGATCCTCTTCAGAAGAAAAT-mgb |
|  | *FAM228B*-E4-R | ATTCCCCTTGCCTCC |  |  |
| 50 | *FAM228B*-E6-F | GTTACCATCCCACCATT | 80 |  |
|  | *FAM228B*-E6-R | CACTGAAGAAGAGTTCTTTT |  |  |
| 51 | *FAM228B*-E7-F | GCATTCCAGATTCCCA | 105 | fam-CTTGTAGGCAGTTTCAGCCACT-mgb |
|  | *FAM228B*-E7-R | CTCCTTCTACAAAATTCACTT |  |  |
| 52 | *NCF2*-E3-F | ATATGATTTGGCTATCAAAGACC | 106 |  |
|  | *NCF2*-E3-R | ACAGGCAAACAGCTTGAACT |  |  |
| 53 | *NCF2*-E5-F | CCAGTGGTGATCCCTGTGG | 90 |  |
|  | *NCF2*-E5-R | CGTCGCCTTGCCTAGGTAAT |  |  |
| 54 | *NCF2*-E15-F | AAGAATGGCTGGAAGGGGAGT | 94 | vic-CATTTTCCCCAAAGTTTTTGTT-mgb |
|  | *NCF2*-E15-R | CGAGTGCTTTCCAAATCTGTAGTT |  |  |
| 55 | *SRSF2*-E1-F | CCCGCCCAGTTGTTACTCAGGT | 92 | vic-CTAGCCTGCGGAGCCCGT-mgb |
|  | *SRSF2*-E1-R | GCCTTCCGCGTGGGGACACT |  |  |
| 56 | *SRSF2*-E2-F | AAGTCCAAGTCCTCGTCGGT | 103 |  |
|  | *SRSF2*-E2-R | ACCTGGATTTGGATTCCCTCT |  |  |
| 57 | *SRSF2*-E3-F | CAGAGTGCTTGGCTGTTTCC | 111 | vic-CTCCCGATTGCTCCTGTGTA-mgb |
|  | *SRSF2*-E3-R | AAGTGCAGTTGTCAGGCATTTTA |  |  |
| 58 | *FOXA1*-Full length-F | ATGTTAGGAACTGTGAAGATGGAA | 1419 |  |
|  | *FOXA1*-Full length-R | CTAGGAAGTGTTTAGGACGGGTC |  |  |
| 59 | *AR-*Full length-F | ATGGAAGTGCAGTTAGGGCT | 2763 |  |
|  | *AR-*Full length-R | TCACTGGGTGTGGAAATAGATG |  |  |
| 60 | *KLK3-*E1-F | AGCCCCAAGCTTACCACC | 62 |  |
|  | *KLK3-*E1-R | GAAGACAACCGGGACCCA |  |  |
| 61 | *KLK3*-E2-F | TCTCGGATTGTGGGAGGC | 82 |  |
|  | *KLK3*-E2-R | AGACTGCCCTGCCACGAG |  |  |
| 62 | *KLK3*-E3-F | AGCGTGATCTTGCTGGGTC | 79 |  |
|  | *KLK3*-E3-R | GGAAGCTGTGGCTGACCT |  |  |
| 63 | *KLK3*-E4-F | TGTGGACCTCCATGTTATTTCC | 93 |  |
|  | *KLK3*-E4- R | CCTGTCCAGCGTCCAGCA |  |  |
| 64 | *KLK3*-E5-F | GGTATCACGTCATGGGGCAGT | 104 |  |
|  | *KLK3*-E5- R | GCCACGATGGTGTCCTTG |  |  |
| 65 | *KLK3-Full length-F/R* | ATGTGGGTCCCGGTTGTC | 872 |  |
|  | *KLK3-Full length-F/R* | TCAGGGGTTGGCCACGA |  |  |

**Additional File 1: Table S3** The list of upregulated emRNA in PCa

| Transcript_id | gene_name | pvalue | Log2FoldChange |
| --- | --- | --- | --- |
| ENST00000434813 | CLK1 | 0.01608654 | 3.879019874 |
| ENST00000317058 | SGF29 | 0.0210449 | 3.62958893 |
| ENST00000312053 | ADGRE1 | 0.02676547 | 3.579695917 |
| ENST00000594493 | RPS11 | 0.03689065 | 3.38894457 |
| ENST00000359995 | SRSF2 | 0.04153946 | 3.337079377 |
| ENST00000371674 | UBE2V1 | 0.02806166 | 3.241182189 |
| ENST00000548580 | MYL6 | 0.04437032 | 3.2169168 |
| ENST00000223500 | CHMP5 | 0.02200349 | 3.213867017 |
| ENST00000260743 | CALHM2 | 0.0335791 | 3.006447477 |
| ENST00000553300 | HNRNPC | 0.02840453 | 2.944468369 |
| ENST00000318887 | C3orf38 | 0.03313877 | 2.90505314 |
| ENST00000269391 | RNF157 | 0.0498394 | 2.7882788 |
| ENST00000371717 | PMPCA | 0.04718546 | 2.744420383 |
| ENST00000362006 | CCDC7 | 0.03586922 | 2.738599741 |
| ENST00000367535 | NCF2 | 0.03368402 | 2.732805914 |
| ENST00000552056 | NAP1L1 | 0.03872763 | 2.70331052 |
| ENST00000265245 | LSG1 | 0.03028411 | 2.676025286 |
| ENST00000226004 | DUSP3 | 0.02370094 | 2.656856326 |
| ENST00000240079 | CCDC53 | 0.02868969 | 2.625278381 |
| ENST00000615575 | FAM228B | 0.009739085 | 2.615939849 |
| ENST00000525643 | IL32 | 0.002323447 | 2.577826263 |
| ENST00000425134 | TXNIP | 0.02749083 | 2.515888383 |
| ENST00000338639 | PARK7 | 0.04520649 | 2.47891633 |
| ENST00000277632 | FAM188A | 0.01205536 | 2.393481269 |
| ENST00000367088 | DYNLT1 | 0.0338962 | 2.38423018 |
| ENST00000402802 | PDGFA | 0.0282031 | 2.380888593 |
| ENST00000250092 | CD68 | 0.04513267 | 2.297208499 |
| ENST00000276689 | NDUFB9 | 0.04018359 | 2.281334859 |
| ENST00000406246 | RELA | 0.04574384 | 2.268520869 |
| ENST00000358855 | NOL8 | 0.02803342 | 2.25163263 |
| ENST00000521389 | RNF130 | 0.02867853 | 2.250361014 |
| ENST00000307641 | NKIRAS2 | 0.04838566 | 2.247818143 |
| ENST00000392870 | GRK5 | 0.01699537 | 2.244614749 |
| ENST00000399220 | CX3CR1 | 0.03425869 | 2.231734166 |
| ENST00000321301 | TOMM5 | 0.03646213 | 2.201015689 |
| ENST00000227155 | CD82 | 0.04838149 | 2.182455652 |
| ENST00000221957 | PLIN3 | 0.03012861 | 2.182230657 |
| ENST00000586839 | AES | 0.02773433 | 2.180625364 |
| ENST00000616356 | FCN1 | 0.01720628 | 2.178769235 |
| ENST00000488423 | DPH3 | 0.0309113 | 2.157498187 |
| ENST00000574081 | SCIMP | 0.02286578 | 2.142380838 |
| ENST00000184266 | NDUFB4 | 0.03968992 | 2.128977267 |
| ENST00000392920 | EVL | 0.04583117 | 2.121803146 |
| ENST00000257818 | LMO2 | 0.0120522 | 2.117359013 |
| ENST00000418388 | C9orf69 | 0.02555291 | 2.116842526 |
| ENST00000317096 | PARL | 0.03038647 | 2.094630326 |
| ENST00000607357 | MYEOV2 | 0.006531488 | 2.089672027 |
| ENST00000296417 | H2AFZ | 0.005536707 | 2.082228737 |
| ENST00000287156 | UBE2L6 | 0.01652695 | 2.057039037 |
| ENST00000592138 | TPM4 | 0.02870339 | 2.029428847 |
| ENST00000444129 | RECQL | 0.01905763 | 2.028109592 |
| ENST00000264316 | TXK | 0.01693774 | 2.003867796 |
| ENST00000300249 | MAPRE2 | 0.008243421 | 2.001561413 |
| ENST00000353107 | POLR2K | 0.02665378 | 1.996592911 |
| ENST00000373547 | PPP6C | 0.03024893 | 1.975527632 |
| ENST00000559199 | SPG21 | 0.004809912 | 1.965808067 |
| ENST00000534025 | TMEM9B | 0.01654052 | 1.956923416 |
| ENST00000220669 | ZFAND1 | 0.03204032 | 1.956363639 |
| ENST00000401827 | PDE7A | 0.02016491 | 1.93884623 |
| ENST00000222008 | RABAC1 | 0.04089321 | 1.935612117 |
| ENST00000375897 | ACBD5 | 0.03014603 | 1.927711875 |
| ENST00000322054 | EHD3 | 0.03485725 | 1.926335343 |
| ENST00000349048 | PFKL | 0.01902878 | 1.907083991 |
| ENST00000528610 | EEF1D | 0.01546764 | 1.88163667 |
| ENST00000301964 | TADA3 | 0.001990471 | 1.877850943 |
| ENST00000577571 | RASSF5 | 0.00352397 | 1.849463757 |
| ENST00000367495 | RAB32 | 0.02187216 | 1.826319264 |
| ENST00000267202 | VPS37B | 0.002702464 | 1.808940749 |
| ENST00000503008 | 43160 | 0.02388341 | 1.803189369 |
| ENST00000415914 | THAP5 | 0.03351881 | 1.787629433 |
| ENST00000396290 | ID2 | 0.01932569 | 1.785858121 |
| ENST00000271764 | EIF2D | 0.04075835 | 1.766476315 |
| ENST00000623998 | CH507-9B2.9 | 0.04220312 | 1.765014596 |
| ENST00000263097 | CNN2 | 0.004831046 | 1.754759844 |
| ENST00000352645 | ZC3H7B | 0.03064344 | 1.735409428 |
| ENST00000358402 | MAX | 0.01971786 | 1.735293793 |
| ENST00000405356 | NOLC1 | 0.04865618 | 1.726847139 |
| ENST00000413699 | RPL15 | 0.01377791 | 1.720452428 |
| ENST00000369769 | KCNA3 | 0.04575832 | 1.716947688 |
| ENST00000234160 | GORASP2 | 0.01702115 | 1.705868766 |
| ENST00000290573 | HK2 | 0.04861216 | 1.694035979 |
| ENST00000357743 | GOLGA7 | 0.03972414 | 1.685697432 |
| ENST00000262629 | TYROBP | 0.02721713 | 1.675862604 |
| ENST00000378551 | PPM1B | 0.01662829 | 1.665613602 |
| ENST00000375151 | TSR2 | 0.04218712 | 1.655960003 |
| ENST00000285930 | AKR1B1 | 0.03112566 | 1.639685642 |
| ENST00000320486 | ERCC6L2 | 0.03884272 | 1.638557622 |
| ENST00000216129 | TTLL12 | 0.03496152 | 1.635141143 |
| ENST00000303221 | EMB | 0.003447325 | 1.633084615 |
| ENST00000322753 | MINOS1 | 0.03265274 | 1.616205399 |
| ENST00000263864 | VAMP8 | 0.03864844 | 1.615772743 |
| ENST00000259873 | MRPS18B | 0.04084058 | 1.600817969 |
| ENST00000297290 | BRI3 | 0.01620363 | 1.595173292 |
| ENST00000431877 | SSFA2 | 0.002730255 | 1.595052529 |
| ENST00000549920 | RPL18 | 0.01092124 | 1.592817114 |
| ENST00000399598 | UBXN2B | 0.004951329 | 1.586263822 |
| ENST00000348943 | HNRNPM | 0.01972528 | 1.576881151 |
| ENST00000261267 | LYZ | 0.009094818 | 1.567643079 |
| ENST00000338981 | USP9Y | 0.03667932 | 1.556809213 |
| ENST00000479870 | TCAF1 | 0.03886805 | 1.555758259 |
| ENST00000352690 | CD96 | 0.04007948 | 1.549705181 |
| ENST00000285279 | VOPP1 | 0.03667744 | 1.540529248 |
| ENST00000379400 | RASSF2 | 0.005443776 | 1.53362941 |
| ENST00000263863 | GNLY | 0.04717057 | 1.531906221 |
| ENST00000374980 | EIF2S2 | 0.003378856 | 1.529046113 |
| ENST00000303391 | MECP2 | 0.009360843 | 1.527983695 |
| ENST00000359520 | TECPR2 | 0.04088506 | 1.521440088 |
| ENST00000521180 | ST3GAL1 | 0.03466086 | 1.516732302 |
| ENST00000353245 | YWHAZ | 0.04280528 | 1.512399532 |
| ENST00000305264 | HDAC3 | 0.03378099 | 1.508569334 |
| ENST00000276420 | DOK2 | 0.01282296 | 1.504170667 |
| ENST00000344229 | RNF13 | 0.01500789 | 1.490009566 |
| ENST00000303904 | COPS6 | 0.04736206 | 1.477129937 |
| ENST00000596831 | AC004076.9 | 0.03684145 | 1.475699205 |
| ENST00000397147 | NCF4 | 0.04192652 | 1.475156293 |
| ENST00000349995 | COG3 | 0.04894982 | 1.469496103 |
| ENST00000254942 | TERF2 | 0.01740166 | 1.467539442 |
| ENST00000296666 | PRRC1 | 0.03854835 | 1.466943928 |
| ENST00000274364 | IQGAP2 | 0.00390224 | 1.463608766 |
| ENST00000433473 | PPT1 | 0.04070716 | 1.462020447 |
| ENST00000263694 | SNRNP40 | 0.01781331 | 1.459595235 |
| ENST00000344548 | CDC42 | 0.007125902 | 1.453781065 |
| ENST00000324679 | SAV1 | 0.02499445 | 1.451645367 |
| ENST00000357156 | DYNLRB1 | 0.02900395 | 1.448122063 |
| ENST00000261517 | VPS13C | 0.02696515 | 1.447590124 |
| ENST00000278616 | ATM | 0.006688717 | 1.43911349 |
| ENST00000267938 | UBE2Q2 | 0.01663798 | 1.436692819 |
| ENST00000408965 | CEBPD | 0.01801617 | 1.433447775 |
| ENST00000349792 | PIP5K1A | 0.04740031 | 1.42154337 |
| ENST00000422843 | ITK | 0.02931978 | 1.416118857 |
| ENST00000272519 | RALB | 0.02532647 | 1.40319036 |
| ENST00000556766 | GNG2 | 0.03366374 | 1.400891018 |
| ENST00000515393 | MCTP1 | 0.02079209 | 1.396915183 |
| ENST00000318948 | NRIP1 | 0.02772413 | 1.3955833 |
| ENST00000360128 | MAK16 | 0.03985502 | 1.389998256 |
| ENST00000248598 | FGL2 | 0.009962921 | 1.38788932 |
| ENST00000628286 | BMP2K | 0.01978993 | 1.385524114 |
| ENST00000369681 | CYB5R4 | 0.02896677 | 1.384814041 |
| ENST00000395310 | SEC31A | 0.003013353 | 1.371829775 |
| ENST00000278618 | AASDHPPT | 0.04169833 | 1.370989038 |
| ENST00000238081 | YWHAQ | 0.01310029 | 1.364985179 |
| ENST00000380636 | TMSB4X | 0.005755807 | 1.362223927 |
| ENST00000346234 | OSTF1 | 0.003947223 | 1.340679917 |
| ENST00000392869 | GTDC1 | 0.008044158 | 1.33275068 |
| ENST00000396591 | SLC30A5 | 0.04270541 | 1.329026883 |
| ENST00000358290 | HSD17B11 | 0.007242594 | 1.323724577 |
| ENST00000511367 | DROSHA | 0.04171727 | 1.323223048 |
| ENST00000330714 | MX2 | 0.01862386 | 1.310016017 |
| ENST00000350669 | LDHB | 0.01392431 | 1.305716534 |
| ENST00000216714 | APEX1 | 0.04420153 | 1.298366171 |
| ENST00000373855 | CNTRL | 0.01276737 | 1.294408182 |
| ENST00000263347 | TCF25 | 0.03101013 | 1.289944579 |
| ENST00000605042 | FBXW7 | 0.03686597 | 1.289727199 |
| ENST00000322989 | RPS15A | 0.0301887 | 1.286703125 |
| ENST00000329235 | AP1S2 | 0.002633366 | 1.285956021 |
| ENST00000377615 | COMMD6 | 0.03277703 | 1.279415085 |
| ENST00000372409 | PCIF1 | 0.03253416 | 1.271939693 |
| ENST00000423759 | TAF1 | 0.04418621 | 1.270809703 |
| ENST00000617275 | PLEKHA2 | 0.01755429 | 1.270434001 |
| ENST00000311672 | UQCRH | 0.01627885 | 1.269580496 |
| ENST00000368985 | CTSS | 0.01217848 | 1.266627866 |
| ENST00000263464 | BIRC3 | 0.03928238 | 1.266110909 |
| ENST00000256935 | DOCK2 | 0.00302247 | 1.259690518 |
| ENST00000431828 | KANSL3 | 0.02718898 | 1.258965354 |
| ENST00000519374 | ATP6V0E1 | 0.003525285 | 1.2568035 |
| ENST00000427738 | CYLD | 0.02083528 | 1.256781398 |
| ENST00000397938 | EWSR1 | 0.0191864 | 1.255138576 |
| ENST00000216044 | GTPBP1 | 0.03298091 | 1.254395486 |
| ENST00000281631 | PARP8 | 0.01524051 | 1.251921555 |
| ENST00000395386 | PHF20L1 | 0.02716465 | 1.251300154 |
| ENST00000286788 | CCT8 | 0.01684385 | 1.251092215 |
| ENST00000370192 | DPYD | 0.01299844 | 1.250831968 |
| ENST00000369103 | RGS10 | 0.03497622 | 1.245755421 |
| ENST00000310118 | PSMD2 | 0.02291456 | 1.245684107 |
| ENST00000319074 | C14orf119 | 0.04627315 | 1.240621413 |
| ENST00000450295 | SEMA4D | 0.03771401 | 1.238574801 |
| ENST00000611185 | RNF145 | 0.04945131 | 1.233177489 |
| ENST00000360830 | RPS24 | 0.005625412 | 1.225652916 |
| ENST00000231572 | RARS | 0.03345719 | 1.224377164 |
| ENST00000234313 | PLEK | 0.001617681 | 1.223615259 |
| ENST00000613569 | TOX4 | 0.04470406 | 1.221657839 |
| ENST00000398805 | HSDL2 | 0.04003625 | 1.216229994 |
| ENST00000271620 | PRUNE | 0.04618801 | 1.213121696 |
| ENST00000328649 | CIB1 | 0.02813624 | 1.211708244 |
| ENST00000426216 | ATP9B | 0.01794882 | 1.208578384 |
| ENST00000367103 | MAPKAPK2 | 0.01368299 | 1.206197816 |
| ENST00000243501 | PLA2G12A | 0.02395333 | 1.204154291 |
| ENST00000342756 | SRP72 | 0.015939 | 1.200909362 |
| ENST00000378588 | CYBB | 0.008198315 | 1.197768579 |
| ENST00000277865 | GLUD1 | 0.007906075 | 1.195720098 |
| ENST00000303115 | IL7R | 0.01696527 | 1.19412486 |
| ENST00000615107 | BIN2 | 0.003342463 | 1.192798734 |
| ENST00000264192 | CYTIP | 0.002490277 | 1.19200309 |
| ENST00000375784 | CLIC1 | 0.03055374 | 1.190619984 |
| ENST00000409645 | MGAT5 | 0.04158298 | 1.18931425 |
| ENST00000596731 | WDR83OS | 0.01350002 | 1.186024381 |
| ENST00000335181 | PKM | 0.04497273 | 1.182598401 |
| ENST00000559916 | B2M | 0.03778383 | 1.182384199 |
| ENST00000295955 | RPL9 | 0.03496273 | 1.178821881 |
| ENST00000336083 | RAB6A | 0.03497971 | 1.178353583 |
| ENST00000316594 | HNRNPH2 | 0.02464667 | 1.17629294 |
| ENST00000370339 | YTHDF1 | 0.01708377 | 1.175759873 |
| ENST00000306065 | ANKRD27 | 0.0428106 | 1.1748664 |
| ENST00000371095 | GNAS | 0.01515274 | 1.174623642 |
| ENST00000272102 | ARF1 | 0.004712413 | 1.172642843 |
| ENST00000351842 | USP4 | 0.01401009 | 1.172476398 |
| ENST00000358435 | TOMM7 | 0.03367981 | 1.168930802 |
| ENST00000315717 | ARPC2 | 0.01858781 | 1.168017855 |
| ENST00000263697 | DNAJC8 | 0.04703061 | 1.167404929 |
| ENST00000259939 | RNF144B | 0.04450288 | 1.160429274 |
| ENST00000238497 | VPS4B | 0.02674502 | 1.159043998 |
| ENST00000303965 | ARAP2 | 0.01001722 | 1.156519108 |
| ENST00000278671 | LAMTOR1 | 0.03771753 | 1.155369944 |
| ENST00000397128 | PRKAA1 | 0.01239282 | 1.15406498 |
| ENST00000282516 | NIPBL | 0.007345885 | 1.151490884 |
| ENST00000610426 | NSA2 | 0.04331459 | 1.150760036 |
| ENST00000263246 | PACSIN2 | 0.03086479 | 1.146822961 |
| ENST00000501597 | RPL41 | 0.008702773 | 1.14120933 |
| ENST00000372806 | STK4 | 0.003944627 | 1.137847011 |
| ENST00000543796 | RAN | 0.04121644 | 1.132390433 |
| ENST00000613507 | FAM65B | 0.003724988 | 1.12974257 |
| ENST00000250617 | ARHGEF6 | 0.01861225 | 1.128685978 |
| ENST00000375847 | DNAJC16 | 0.03862802 | 1.126474109 |
| ENST00000231948 | CRBN | 0.02956305 | 1.12495561 |
| ENST00000295095 | ARHGAP15 | 0.04730175 | 1.124785588 |
| ENST00000247655 | COX7C | 0.02439035 | 1.120332109 |
| ENST00000395468 | MAPK1IP1L | 0.0130823 | 1.118674791 |
| ENST00000338087 | SLA | 0.03671274 | 1.118309099 |
| ENST00000285419 | TMEM55A | 0.04660872 | 1.111205243 |
| ENST00000295321 | IWS1 | 0.04557523 | 1.110293713 |
| ENST00000379359 | RGCC | 0.04296267 | 1.110039504 |
| ENST00000380494 | COL4A3BP | 0.04765121 | 1.109871863 |
| ENST00000381989 | PARP4 | 0.00720208 | 1.106257361 |
| ENST00000378536 | SKI | 0.01870953 | 1.106037348 |
| ENST00000229402 | KLRB1 | 0.04826346 | 1.105978554 |
| ENST00000369252 | ABLIM1 | 0.01506172 | 1.102798123 |
| ENST00000367669 | RFWD2 | 0.04865673 | 1.102515302 |
| ENST00000311417 | ZMAT3 | 0.0173786 | 1.101993236 |
| ENST00000622186 | SLC6A6 | 0.04928065 | 1.100925419 |
| ENST00000378004 | ARHGAP26 | 0.03124261 | 1.098856607 |
| ENST00000261700 | C14orf166 | 0.04348496 | 1.094445915 |
| ENST00000219473 | USP10 | 0.01689069 | 1.094152295 |
| ENST00000543473 | SUDS3 | 0.02525907 | 1.093427152 |
| ENST00000395422 | CHCHD2 | 0.03233121 | 1.088322757 |
| ENST00000281142 | SCLT1 | 0.01153291 | 1.087531464 |
| ENST00000268296 | ITGAX | 0.04500416 | 1.086129213 |
| ENST00000544484 | CHD4 | 0.01154299 | 1.079548132 |
| ENST00000277141 | TUT7 | 0.00974023 | 1.077790612 |
| ENST00000396930 | KAT6A | 0.02914686 | 1.077546897 |
| ENST00000375754 | SYK | 0.04010751 | 1.074311157 |
| ENST00000381334 | TMEM165 | 0.03579313 | 1.071987007 |
| ENST00000314583 | HCLS1 | 0.006891182 | 1.069444913 |
| ENST00000580261 | RPL17 | 0.007794161 | 1.055328242 |
| ENST00000374466 | CSGALNACT2 | 0.02148586 | 1.054534885 |
| ENST00000267205 | RHOF | 0.02106769 | 1.052517778 |
| ENST00000320631 | EHD1 | 0.01781936 | 1.051569588 |
| ENST00000351578 | FYB | 0.004918324 | 1.050277233 |
| ENST00000379066 | PRKD3 | 0.04841416 | 1.048956907 |
| ENST00000399503 | MAP3K1 | 0.00668176 | 1.047912758 |
| ENST00000444313 | PARVG | 0.03816858 | 1.047117733 |
| ENST00000357640 | DENND2D | 0.02650511 | 1.046987333 |
| ENST00000230895 | DAP | 0.01778453 | 1.046443874 |
| ENST00000282272 | ANKRD44 | 0.01328705 | 1.044150104 |
| ENST00000334478 | PFDN5 | 0.01253141 | 1.040620955 |
| ENST00000280098 | SPOPL | 0.02847178 | 1.040424822 |
| ENST00000294383 | USP24 | 0.0240649 | 1.0401207 |
| ENST00000374358 | TMEM50A | 0.03922802 | 1.038524457 |
| ENST00000424459 | GPBP1 | 0.02371025 | 1.037340114 |
| ENST00000529006 | KDM2A | 0.02569982 | 1.036008837 |
| ENST00000554227 | SNRPN | 0.04149868 | 1.034900701 |
| ENST00000294189 | RPL29 | 0.01211768 | 1.034825522 |
| ENST00000290037 | SEC16A | 0.0408685 | 1.031679567 |
| ENST00000293842 | RPL26 | 0.01336191 | 1.031242959 |
| ENST00000262126 | ANKRD12 | 0.01270739 | 1.026627872 |
| ENST00000341776 | JARID2 | 0.02680128 | 1.019953729 |
| ENST00000240185 | TARDBP | 0.04763952 | 1.019698398 |
| ENST00000330899 | DNAJA1 | 0.02304531 | 1.014741396 |
| ENST00000303204 | PRELID1 | 0.02201341 | 1.002765086 |
| ENST00000245932 | VASP | 0.02878718 | 1.002698899 |
| ENST00000510413 | LRBA | 0.009785796 | 1.002069957 |
| ENST00000619426 | PSMB3 | 0.04433995 | 1.000620152 |

**Additional File 1: Table S4** Diagnosis performance of emRNAs

| Variables | Normal and PCa patients | All patients |
| --- | --- | --- |
|  | AUC(95%CI); *P* | AUC(95%CI) ; *P* |
| *CDC42* | 0.857(0.795 to 0.906)  <0.0001 | 0.804(0.757 to 0.848)  <0.0001 |
| *IL32* | 0.824(0.759 to 0.878)  <0.0001 | 0.815(0.767 to 0.856)  <0.0001 |
| *MAX* | 0.707(0.633-0.774)  <0.0001 | 0.754(0.702 to 0.801)  <0.0001 |
| *NCF2* | 0.800(0.732 to 0.857)  <0.0001 | 0.754(0.701 to 0.800)  <0.0001 |
| *PDGFA* | 0.887(0.829 to 0.930)  <0.0001 | 0.689(0.634 to 0.740)  <0.0001 |
| *SRSF2* | 0.740(0.688-0.804)  <0.0001 | 0.786(0.736 to 0.830)  0.0002 |
| emRNAs | 0.948(0.903 to 0.976)  <0.0001 | 0.851(0.806 to 0.888)  <0.0001 |

PCa: prostate cancer; AUC: area under curve; CI: confidence interval; emRNA: exosomal mRNA.

|  |  |  |  |  |  |
| --- | --- | --- | --- | --- | --- |
|  |  |  |  |  |  |
|  |  |  |  |  |  |
|  |  |  |  |  |  |
|  |  |  |  |  |  |
|  |  |  |  |  |  |
|  |  |  |  |  |  |
|  |  |  |  |  |  |
|  |  |  |  |  |  |
|  |  |  |  |  |  |
|  |  |  |  |  |  |
|  |  |  |  |  |  |
|  |  |  |  |  |  |
|  |  |  |  |  |  |
|  |  |  |  |  |  |
|  |  |  |  |  |  |
|  |  |  |  |  |  |
|  |  |  |  |  |  |
|  |  |  |  |  |  |
|  |  |  |  |  |  |
|  |  |  |  |  |  |
|  |  |  |  |  |  |
|  |  |  |  |  |  |
|  |  |  |  |  |  |
|  |  |  |  |  |  |
|  |  |  |  |  |  |
|  |  |  |  |  |  |

**Additional File 1: Table S5** Demographics of PCa patients and control participants for QC of exosome isolation

| No. | Age | Preoperative PSA | G1 | G2 | Gleason Score | Stage | Metastasis | **Treatment** | **CK5/6** | | **P504S** | **PSMA** | **PSAP** |
| --- | --- | --- | --- | --- | --- | --- | --- | --- | --- | --- | --- | --- | --- |
| BPH Patients |  |  |  |  |  |  |  |  | |  |  |  |  |
| 1 | 50 | 16.3 | / |  |  | / | / |  |  | |  |  |  |
| 2 | 69 | 1.153 | / |  |  | / | / |  |  | |  |  |  |
| 3 | 64 | NA | / |  |  | / | / |  |  | |  |  |  |
| 4 | 68 | 74.877 | / |  |  | / | / |  |  | |  |  |  |
| 5 | 70 | 7.37 | / |  |  | / | / |  |  | |  |  |  |
| 6 | 76 | 5.011 | / |  |  | / | / |  |  | |  |  |  |
| 7 | 50 | 16.3 | / |  |  | / | / |  |  | |  |  |  |
| 8 | 69 | 1.153 | / |  |  | / | / |  |  | |  |  |  |
| 9 | 69 | 9.762 | / |  |  | / | / |  |  | |  |  |  |
| 10 | 50 | 16.3 | / |  |  | / | / |  |  | |  |  |  |
| 11 | 61 | 8.584 | / |  |  | / | / |  |  | |  |  |  |
| 12 | 69 | 1.153 | / |  |  | / | / |  |  | |  |  |  |
| PCa patients |  |  |  |  |  |  |  |  |  | |  |  |  |
| 13 | 60 | 5.57 | 4 | 4 | 8 | pT2aN0Mx | 0 | naïve | - | | + | + | + |
| 14 | 67 | 14.95 | 4 | 3 | 7 | pT3aNxMx | 0 | naïve | - | | + | + | + |
| 15 | 62 | 88.34 | 4 | 5 | 9 | pT3aNxMx | 0 | naïve | NA | | NA | NA | NA |
| 16 | 64 | 100 | 4 | 4 | 8 | pT2aN0M0 | 0 | naïve | - | | + | + | + |
| 17 | 61 | 8.26 | 3 | 4 | 7 | pT2aN0M0 | 0 | naïve | - | | + | + | + |
| 18 | 60 | 5.57 | 4 | 4 | 8 | pT2aN0Mx | 0 | naïve | - | | + | + | + |
| 19 | 67 | 14.95 | 4 | 3 | 7 | pT3aNxMx | 0 | naïve | - | | + | + | + |
| 20 | 62 | 88.34 | 4 | 5 | 9 | pT3aNxMx | 1 | naïve | - | | + | + | + |
| 21 | 68 | 14.538 | 4 | 4 | 8 | pT2cNxMx | 0 | naïve | - | | + | + | + |
| 22 | 67 | 14.95 | 4 | 3 | 7 | pT3aNxMx | 0 | naïve | NA | | NA | NA | NA |
| 23 | 60 | 5.57 | 4 | 4 | 8 | pT2aN0Mx | 0 | naïve | NA | | NA | NA | NA |
| 24 | 74 | 222.294 | 4 | 5 | 9 | pT3bN1M1b | 1 | naïve | - | | + | + | + |

|  |  |  |  |
| --- | --- | --- | --- |
|  |  |  |  |
|  |  |  |  |
|  |  |  |  |
|  |  |  |  |
|  |  |  |  |
|  |  |  |  |
|  |  |  |  |
|  |  |  |  |
|  |  |  |  |
|  |  |  |  |
|  |  |  |  |
|  |  |  |  |
|  |  |  |  |
|  |  |  |  |
|  |  |  |  |
|  |  |  |  |
|  |  |  |  |
|  |  |  |  |
|  |  |  |  |
|  |  |  |  |
|  |  |  |  |
|  |  |  |  |
|  |  |  |  |
|  |  |  |  |
|  |  |  |  |
|  |  |  |  |
|  |  |  |  |
|  |  |  |  |
|  |  |  |  |
|  |  |  |  |
|  |  |  |  |
|  |  |  |  |
|  |  |  |  |
|  |  |  |  |
|  |  |  |  |
|  |  |  |  |
|  |  |  |  |
|  |  |  |  |
|  |  |  |  |
|  |  |  |  |
|  |  |  |  |
|  |  |  |  |
|  |  |  |  |
|  |  |  |  |
|  |  |  |  |
|  |  |  |  |
|  |  |  |  |
|  |  |  |  |
|  |  |  |  |
|  |  |  |  |
|  |  |  |  |
|  |  |  |  |
|  |  |  |  |

**Additional File 1: Table S6** Demographics of PCa patients and control participants for RNA-seq of their serum exosome

| **No.** | **Age** | **Preoperative PSA** | **G1** | **G2** | **Gleason socre** | **Stage** | **treatment** | **Metastasis** | **CK5/6** | **P504S** | **PSMA** | **PSAP** |
| --- | --- | --- | --- | --- | --- | --- | --- | --- | --- | --- | --- | --- |
| PCa Patients |  |  |  |  |  |  |  |  |  |  |  |  |
| 1 | 70 | 54.58 | 4 | 5 | 9 | pT3aN0Mx | naïve | 0 | NA | NA | NA | NA |
| 2 | 70 | 8.857 | 3 | 3 | 6 | pT2cN0Mx | naïve | 0 | - | + | + | + |
| 3 | 65 | 26.27 | 5 | 5 | 10 | pT3aN0Mx | naïve | 0 | NA | NA | NA | NA |
| 4 | 67 | 25.769 | 4 | 4 | 8 | pT3aN0Mx | naïve | 0 | NA | NA | NA | NA |
| 5 | 69 | 21.1 | 4 | 4 | 8 | pT3bN0Mx | naïve | 0 | NA | NA | NA | NA |
| 6 | 73 | 62.25 | 4 | 5 | 9 | pT3bN1Mx | naïve | 0 | - | + | + | + |
| 7 | 60 | 20.161 | 3 | 4 | 7 | pT2cN0Mx | naïve | 0 | - | + | + | + |
| 8 | 70 | 37.19 | 4 | 5 | 9 | pT3aN0Mx | naïve | 0 | - | + | + | + |
| 9 | 74 | 88.09 | 4 | 4 | 8 | pT3bN0Mx | naïve | 0 | - | + | + | ± |
| 10 | 63 | 5.469 | 4 | 4 | 8 | pT2cN0Mx | naïve | 0 | - | + | + | + |
| 11 | 75 | 16.48 | 4 | 3 | 10 | pT2aNxMx | naïve | 0 | - | + | + | - |
| 12 | 65 | 37.33 | 4 | 3 | 7 | pT3bN0Mx | naïve | 0 | - | + | + | + |
| 13 | 54 | 38.41 | 4 | 3 | 7 | pT3bNxMx | naïve | 0 | - | + | + | + |
| 14 | 65 | 25.65 | 5 | 4 | 9 | pT3bN0Mx | naïve | 0 | - | + | + | + |
| 15 | 78 | 32 | 4 | 4 | 8 | pT3bN0Mx | naïve | 0 | - | + | + | + |
| 16 | 63 | 46.8 | 4 | 5 | 9 | pT3bN0Mx | naïve | 0 | - | + | + | + |
| 17 | 69 | 9.7 | 5 | 4 | 9 | pT3aN0Mx | naïve | 0 | - | + | + | + |
| 18 | 61 | 10.398 | 4 | 4 | 8 | pT2cN0Mx | naïve | 0 | NA | NA | NA | NA |
| 19 | 66 | 6.78 | 3 | 4 | 7 | pT2aNxMx | naïve | 0 | Basal cell+ | + | + | + |
| 20 | 68 | 6.226 | 3 | 3 | 6 | pT2aN0Mx | naïve | 0 | NA | NA | NA | NA |
| 21 | 70 | 7.3 | 3 | 3 | 6 | pT2bNxMx | naïve | 0 | NA | NA | NA | NA |
| 22 | 66 | 5.187 | 3 | 3 | 6 | pT2cNxMx | naïve | 0 | NA | NA | NA | NA |
| 23 | 59 | 11.158 | 3 | 3 | 6 | pT3aNxMx | naïve | 0 | NA | NA | NA | NA |
| 24 | 63 | 12.11 | 3 | 3 | 6 | pT2aNxMx | naïve | 0 | - | ± | - | + |
| 25 | 62 | 7.626 | 3 | 3 | 6 | pT2cNxMx | naïve | 0 | NA | NA | NA | NA |
| 26 | 71 | 4.874 | 3 | 3 | 6 | pT2aNxMx | naïve | 0 | - | + | + | NA |
| 27 | 45 | 13.93 | 3 | 3 | 6 | pT2aNxMx | naïve | 0 | - | + | + | - |
| 28 | 67 | 6.637 | 3 | 3 | 6 | pT2cNxMx | naïve | 0 | - | + | + | + |
| 29 | 76 | 21.125 | 4 | 3 | 7 | pT2aN0Mx | naïve | 0 | - | + | + | + |
| 30 | 72 | 8.165 | 3 | 3 | 6 | pT2cNxMx | naïve | 0 | - | + | + | + |
| 31 | 64 | 9.60 | 4 | 3 | 7 | pT3aN0Mx | naïve | 0 | - | + | + | + |
| BPH Individuals |  |  |  |  |  |  |  |  |  |  |  |  |
| 32 | 60 | 6.07 |  |  |  |  |  |  |  |  |  |  |
| 33 | 69 | 5.035 |  |  |  |  |  |  |  |  |  |  |
| 34 | 63 | 5.37 |  |  |  |  |  |  |  |  |  |  |
| 35 | 54 | 5.84 |  |  |  |  |  |  |  |  |  |  |
| 36 | 62 | 5.937 |  |  |  |  |  |  |  |  |  |  |
| 37 | 68 | 6.210 |  |  |  |  |  |  |  |  |  |  |
| 38 | 65 | 6.313 |  |  |  |  |  |  |  |  |  |  |
| 39 | 73 | 4.7 |  |  |  |  |  |  |  |  |  |  |
| 40 | 61 | 8.881 |  |  |  |  |  |  |  |  |  |  |
| 41 | 67 | 12.44 |  |  |  |  |  |  |  |  |  |  |
| 42 | 63 | 8.02 |  |  |  |  |  |  |  |  |  |  |
| 43 | 65 | 12.04 |  |  |  |  |  |  |  |  |  |  |
| 44 | 74 | 5.326 |  |  |  |  |  |  |  |  |  |  |
| 45 | 67 | 9.832 |  |  |  |  |  |  |  |  |  |  |
| 46 | 61 | 5.611 |  |  |  |  |  |  |  |  |  |  |
| 47 | 54 | 8.695 |  |  |  |  |  |  |  |  |  |  |
| 48 | 69 | 4.66 |  |  |  |  |  |  |  |  |  |  |

|  |  |  |
| --- | --- | --- |
|  |  |  |
|  |  |  |
|  |  |  |
|  |  |  |
|  |  |  |
|  |  |  |
|  |  |  |
|  |  |  |
|  |  |  |
|  |  |  |
|  |  |  |
|  |  |  |
|  |  |  |
|  |  |  |
|  |  |  |
|  |  |  |
|  |  |  |
|  |  |  |
|  |  |  |
|  |  |  |
|  |  |  |
|  |  |  |
|  |  |  |
|  |  |  |
|  |  |  |
|  |  |  |
|  |  |  |
|  |  |  |
|  |  |  |
|  |  |  |
|  |  |  |
|  |  |  |
|  |  |  |
|  |  |  |
|  |  |  |
|  |  |  |
|  |  |  |
|  |  |  |
|  |  |  |
|  |  |  |
|  |  |  |
|  |  |  |
|  |  |  |
|  |  |  |
|  |  |  |
|  |  |  |
|  |  |  |
|  |  |  |
|  |  |  |
|  |  |  |
|  |  |  |
|  |  |  |
|  |  |  |
|  |  |  |
|  |  |  |
|  |  |  |
|  |  |  |
|  |  |  |
|  |  |  |
|  |  |  |
|  |  |  |
|  |  |  |
|  |  |  |
|  |  |  |
|  |  |  |
|  |  |  |
|  |  |  |
|  |  |  |
|  |  |  |
|  |  |  |
|  |  |  |
|  |  |  |
|  |  |  |
|  |  |  |
|  |  |  |
|  |  |  |
|  |  |  |
|  |  |  |
|  |  |  |
|  |  |  |
|  |  |  |
|  |  |  |
|  |  |  |
|  |  |  |
|  |  |  |
|  |  |  |
|  |  |  |
|  |  |  |
|  |  |  |
|  |  |  |
|  |  |  |
|  |  |  |
|  |  |  |
|  |  |  |
|  |  |  |
|  |  |  |
|  |  |  |
|  |  |  |
|  |  |  |
|  |  |  |
|  |  |  |
|  |  |  |
|  |  |  |
|  |  |  |
|  |  |  |
|  |  |  |
|  |  |  |
|  |  |  |
|  |  |  |
|  |  |  |
|  |  |  |
|  |  |  |
|  |  |  |
|  |  |  |
|  |  |  |
|  |  |  |
|  |  |  |
|  |  |  |
|  |  |  |
|  |  |  |
|  |  |  |
|  |  |  |
|  |  |  |
|  |  |  |
|  |  |  |
|  |  |  |
|  |  |  |
|  |  |  |
|  |  |  |
|  |  |  |
|  |  |  |
|  |  |  |
|  |  |  |
|  |  |  |
|  |  |  |
|  |  |  |
|  |  |  |
|  |  |  |
|  |  |  |
|  |  |  |
|  |  |  |
|  |  |  |
|  |  |  |
|  |  |  |
|  |  |  |
|  |  |  |
|  |  |  |
|  |  |  |
|  |  |  |
|  |  |  |
|  |  |  |
|  |  |  |
|  |  |  |
|  |  |  |
|  |  |  |
|  |  |  |
|  |  |  |
|  |  |  |
|  |  |  |
|  |  |  |
|  |  |  |
|  |  |  |
|  |  |  |
|  |  |  |
|  |  |  |
|  |  |  |
|  |  |  |
|  |  |  |
|  |  |  |
|  |  |  |
|  |  |  |
|  |  |  |
|  |  |  |
|  |  |  |
|  |  |  |
|  |  |  |
|  |  |  |
|  |  |  |
|  |  |  |
|  |  |  |
|  |  |  |
|  |  |  |
|  |  |  |
|  |  |  |
|  |  |  |
|  |  |  |
|  |  |  |
|  |  |  |
|  |  |  |
|  |  |  |
|  |  |  |
|  |  |  |
|  |  |  |
|  |  |  |
|  |  |  |
|  |  |  |
|  |  |  |
|  |  |  |
|  |  |  |
|  |  |  |
|  |  |  |
|  |  |  |
|  |  |  |
|  |  |  |
|  |  |  |
|  |  |  |
|  |  |  |
|  |  |  |
|  |  |  |
|  |  |  |
|  |  |  |
|  |  |  |
|  |  |  |
|  |  |  |
|  |  |  |
|  |  |  |
|  |  |  |
|  |  |  |
|  |  |  |
|  |  |  |
|  |  |  |
|  |  |  |
|  |  |  |
|  |  |  |
|  |  |  |
|  |  |  |
|  |  |  |
|  |  |  |
|  |  |  |
|  |  |  |
|  |  |  |
|  |  |  |
|  |  |  |
|  |  |  |
|  |  |  |
|  |  |  |
|  |  |  |
|  |  |  |
|  |  |  |
|  |  |  |
|  |  |  |
|  |  |  |
|  |  |  |
|  |  |  |
|  |  |  |
|  |  |  |
|  |  |  |
|  |  |  |
|  |  |  |
|  |  |  |
|  |  |  |
|  |  |  |
|  |  |  |
|  |  |  |
|  |  |  |
|  |  |  |
|  |  |  |
|  |  |  |
|  |  |  |
|  |  |  |
|  |  |  |
|  |  |  |
|  |  |  |
|  |  |  |
|  |  |  |
|  |  |  |
|  |  |  |
|  |  |  |
|  |  |  |
|  |  |  |
|  |  |  |
|  |  |  |
|  |  |  |
|  |  |  |
|  |  |  |
|  |  |  |
|  |  |  |
|  |  |  |
|  |  |  |
|  |  |  |
|  |  |  |
|  |  |  |
|  |  |  |
|  |  |  |
|  |  |  |
|  |  |  |
|  |  |  |
|  |  |  |
|  |  |  |
|  |  |  |
|  |  |  |
|  |  |  |
|  |  |  |
|  |  |  |
|  |  |  |
|  |  |  |
|  |  |  |
|  |  |  |
|  |  |  |
|  |  |  |
|  |  |  |
|  |  |  |
|  |  |  |
|  |  |  |
|  |  |  |
|  |  |  |
|  |  |  |
|  |  |  |
|  |  |  |
|  |  |  |
|  |  |  |
|  |  |  |
|  |  |  |
|  |  |  |
|  |  |  |
|  |  |  |
|  |  |  |
|  |  |  |
|  |  |  |
|  |  |  |
|  |  |  |
|  |  |  |
|  |  |  |
|  |  |  |
|  |  |  |
|  |  |  |
|  |  |  |
|  |  |  |
|  |  |  |
|  |  |  |
|  |  |  |
|  |  |  |
|  |  |  |
|  |  |  |
|  |  |  |
|  |  |  |
|  |  |  |
|  |  |  |
|  |  |  |
|  |  |  |
|  |  |  |

**Additional File 1: Table S7** Demographcs of PCa patients and control participants for dysregulated emRNAs validation

| Sample Name | Diagnosis | PSA | age | G1 | G2 | Gleason socre | Stage | treatment | Metastasis | CK5/6 | P504S | PSMA | PSAP |
| --- | --- | --- | --- | --- | --- | --- | --- | --- | --- | --- | --- | --- | --- |
| 1 | BPH | 10 | 64 |  |  |  |  |  |  |  |  |  |  |
| 2 | PCA | 10.5 | 67 | 5 | 5 | 10 | pT3bN0Mx | naïve | 0 | - | + | + | + |
| 3 | PCA | 8.91 | 69 | 3 | 3 | 6 | pT2cNxMx | naïve | 0 | - | + | + | + |
| 4 | PCA | 100 | 71 | 4 | 5 | 9 | pT2cN1M1 | naïve | 1 | NA | NA | NA | NA |
| 5 | BPH | 7.37 | 74 |  |  |  |  |  |  |  |  |  |  |
| 6 | BPH | 8.286 | 70 |  |  |  |  |  |  |  |  |  |  |
| 7 | PCA | 25.09 | 67 | 5 | 5 | 10 | pT3bN0Mx | naïve | 0 | - | + | + | + |
| 8 | BPH | 8.2 | 61 |  |  |  |  |  |  |  |  |  |  |
| 9 | BPH | 4 | 53 |  |  |  |  |  |  |  |  |  |  |
| 10 | BPH | 8.93 | 47 |  |  |  |  |  |  |  |  |  |  |
| 11 | BPH | 14.98 | 73 |  |  |  |  |  |  |  |  |  |  |
| 12 | BPH | 37.61 | 53 |  |  |  |  |  |  |  |  |  |  |
| 13 | PCA | 9.5 | 61 | 3 | 4 | 7 | pT2cN0Mx | naïve | 0 | - | + | + | + |
| 14 | BPH | 7.23 | 61 |  |  |  |  |  |  |  |  |  |  |
| 15 | BPH | 10.44 | 67 |  |  |  |  |  |  |  |  |  |  |
| 16 | BPH | 9.289 | 67 |  |  |  |  |  |  |  |  |  |  |
| 17 | PCA | 7.121 | 56 | 3 | 4 | 7 | pT2aNxMx | naïve | 0 | - | + | + | - |
| 19 | BPH | 8.83 | 67 |  |  |  |  |  |  |  |  |  |  |
| 20 | PCA | 38 | 65 | 4 | 4 | 8 | pT3bNxMx | naïve | 1 | - | + | + | + |
| 21 | BPH | 0.94 | 48 |  |  |  |  |  |  |  |  |  |  |
| 22 | BPH | 7.46 | 69 |  |  |  |  |  |  |  |  |  |  |
| 23 | BPH | 12.39 | 70 |  |  |  |  |  |  |  |  |  |  |
| 24 | BPH | 100 | 72 |  |  |  |  |  |  |  |  |  |  |
| 26 | PCA | 4.63 | 58 | 3 | 4 | 7 | pT2aN0Mx | naïve | 0 | - | + | + | + |
| 27 | BPH | 10.19 | 52 |  |  |  |  |  |  |  |  |  |  |
| 28 | BPH | 8.48 | 52 |  |  |  |  |  |  |  |  |  |  |
| 29 | PCA | 10.1 | 76 | 3 | 3 | 6 | pT2cNxMx | naïve | 0 | - | - | + | + |
| 30 | BPH | 6.97 | 69 |  |  |  |  |  |  |  |  |  |  |
| 31 | BPH | 5.491 | 58 |  |  |  |  |  |  |  |  |  |  |
| 32 | PCA | 27.853 | 72 | 4 | 4 | 8 | pT4NxMx | naïve | 1 | - | + | + | + |
| 33 | PCA | 100 | 64 | 4 | 5 | 9 | pT2cNxMx | naïve | 0 | - | + | + | + |
| 34 | PCA | 8.28 | 75 | 3 | 4 | 7 | pT2aNxMx | naïve | 0 | - | + | + | + |
| 35 | PCA | 37.16 | 71 | 4 | 4 | 8 | pT3bNxMx | naïve | 0 | NA | NA | NA | NA |
| 36 | PCA | 88.6 | 70 | 4 | 5 | 9 | pT4N1M1 | naïve | 1 | - | + | + | + |
| 37 | BPH | 5.57 | 69 |  |  |  |  |  |  |  |  |  |  |
| 38 | BPH | 8.997 | 61 |  |  |  |  |  |  |  |  |  |  |
| 40 | BPH | 6.668 | 74 |  |  |  |  |  |  |  |  |  |  |
| 41 | BPH | 6 | 69 |  |  |  |  |  |  |  |  |  |  |
| 42 | BPH | 6.235 | 68 |  |  |  |  |  |  |  |  |  |  |
| 44 | PCA | 100 | 66 | 5 | 4 | 9 | pT2cNxMx | naïve | 0 | - | + | + | + |
| 45 | PCA | 77.42 | 63 | 4 | 4 | 8 | pT2cNxMx | naïve | 0 | - | + | + | + |
| 46 | BPH | 12.58 | 67 |  |  |  |  |  |  |  |  |  |  |
| 47 | BPH | 15 | 55 |  |  |  |  |  |  |  |  |  |  |
| 48 | PCA | 28.06 | 73 | 5 | 4 | 9 | pT3bN1Mx | naïve | 0 | - | + | + | + |
| 49 | BPH | 9 | 66 |  |  |  |  |  |  |  |  |  |  |
| 50 | PCA | 100 | 61 | 5 | 4 | 9 | pT2cNxMx | naïve | 0 | - | + | + | + |
| 51 | PCA | 22.81 | 66 | 4 | 5 | 9 | pT3bNxMx | naïve | 0 | - | + | + | + |
| 52 | PCA | 9.38 | 63 | 4 | 4 | 8 | pT2aNxMx | naïve | 0 | - | + | + | + |
| 54 | PCA | 6.86 | 58 | 3 | 4 | 7 | pT2cNxMx | naïve | 0 | - | + | + | + |
| 55 | BPH | 8.75 | 68 |  |  |  |  |  |  |  |  |  |  |
| 56 | PCA | 10.16 | 69 | 3 | 4 | 7 | pT2cNxMx | naïve | 0 | - | + | + | + |
| 57 | PCA | 100 | 79 | 4 | 4 | 8 | pT2aNxMx | naïve | 0 | - | + | + | + |
| 58 | PCA | 85 | 76 | 5 | 4 | 9 | pT3aN0Mx | naïve | 0 | - | + | + | + |
| 59 | PCA | 100 | 66 | 4 | 5 | 9 | pT3bN1Mx | naïve | 0 | - | + | + | + |
| 60 | PCA | 4 | 72 | 4 | 4 | 8 | pT2cN0Mx | naïve | 0 | - | + | + | + |
| 61 | PCA | 13.96 | 80 | 4 | 4 | 8 | pT3bNxMx | naïve | 0 | - | + | + | + |
| 62 | BPH | 9.353 | 68 |  |  |  |  |  |  |  |  |  |  |
| 63 | PCA | 9.36 | 56 | 4 | 3 | 7 | pT2cNxMx | naïve | 0 | NA | NA | NA | NA |
| 64 | BPH | 6.71 | 62 |  |  |  |  |  |  |  |  |  |  |
| 65 | PCA | 29.256 | 85 | 3 | 4 | 7 | pT2cN0Mx | naïve | 0 | - | + | + | + |
| 66 | PCA | 63.39 | 78 | 4 | 5 | 9 | pT2aNxMx | naïve | 0 | - | + | + | + |
| 67 | PCA | 100 | 71 | 4 | 5 | 9 | pT2cNxMx | naïve | 0 | - | + | + | + |
| 68 | BPH | 0.59 | 41 |  |  |  |  |  |  |  |  |  |  |
| 69 | BPH | 5.46 | 68 |  |  |  |  |  |  |  |  |  |  |
| 71 | BPH | 5.76 | 62 |  |  |  |  |  |  |  |  |  |  |
| 72 | PCA | 16.956 | 62 | 5 | 5 | 10 | pT3bNxMx | naïve | 0 | NA | NA | NA | NA |
| 73 | PCA | 18.01 | 76 | 3 | 4 | 7 | pT2cNxMx | naïve | 0 | - | + | + | + |
| 74 | BPH | 4.65 | 66 |  |  |  |  |  |  |  |  |  |  |
| 75 | PCA | 13 | 70 | 4 | 4 | 8 | pT2cN0Mx | naïve | 1 | - | + | + | + |
| 76 | PCA | 40.214 | 75 | 4 | 4 | 8 | pT3aNxMx | naïve | 0 | - | + | + | + |
| 77 | BPH | 6.57 | 55 |  |  |  |  |  |  |  |  |  |  |
| 78 | BPH | 7.69 | 64 |  |  |  |  |  |  |  |  |  |  |
| 80 | BPH | 19 | 68 |  |  |  |  |  |  |  |  |  |  |
| 81 | PCA | 5.78 | 60 | 3 | 3 | 6 | pT2aNxMx | naïve | 0 | - | + | + | + |
| 82 | PCA | 6.26 | 65 | \ | \ | \ | pT3aNxMx | naïve | 0 | - | + | + | + |
| 83 | PCA | 100 | 67 | 4 | 3 | 7 | pT2cNxMx | naïve | 0 | - | + | + | + |
| 84 | BPH | 11.2 | 59 |  |  |  |  |  |  |  |  |  |  |
| 85 | PCA | 100 | 76 | 5 | 4 | 9 | pT2cNxMx | naïve | 0 | - | + | + | + |
| 86 | BPH | 21 | 70 |  |  |  |  |  |  |  |  |  |  |
| 87 | PCA | 5.61 | 68 | 3 | 3 | 6 | pT2cNxMx | naïve | 0 | - | + | + | + |
| 88 | PCA | 12.953 | 73 | 3 | 4 | 7 | pT2cNxMx | naïve | 0 | - | + | + | + |
| 89 | BPH | 13.312 | 70 |  |  |  |  |  |  |  |  |  |  |
| 91 | BPH | 12.58 | 68 |  |  |  |  |  |  |  |  |  |  |
| 92 | PCA | 11.19 | 57 | 4 | 5 | 9 | pT3aN0Mx | naïve | 0 | - | + | + | + |
| 93 | BPH | 9.113 | 76 |  |  |  |  |  |  |  |  |  |  |
| 94 | PCA | 100 | 68 | 5 | 4 | 9 | pT2cNxMx | naïve | 0 | - | + | + | + |
| 95 | BPH | 11.7 | 64 |  |  |  |  |  |  |  |  |  |  |
| 96 | PCA | 16.67 | 68 | 4 | 3 | 7 | pT2cNxMx | naïve | 0 | - | + | + | + |
| 97 | BPH | 5.96 | 68 |  |  |  |  |  |  |  |  |  |  |
| 98 | BPH | 8.42 | 58 |  |  |  |  |  |  |  |  |  |  |
| 99 | BPH | 15.295 | 61 |  |  |  |  |  |  |  |  |  |  |
| 100 | BPH | 5.82 | 60 |  |  |  |  |  |  |  |  |  |  |
| 101 | BPH | 10.1 | 70 |  |  |  |  |  |  |  |  |  |  |
| 102 | PCA | 4.5 | 66 | 3 | 4 | 7 | pT3aNxMx | naïve | 0 | - | + | + | + |
| 103 | BPH | 13.6 | 50 |  |  |  |  |  |  |  |  |  |  |
| 105 | PCA | 93 | 68 | 5 | 5 | 10 | pT2aNxMx | naïve | 0 | - | + | + | + |
| 106 | PCA | 14.445 | 72 | 3 | 3 | 6 | pT2cN0Mx | naïve | 0 | NA | NA | NA | NA |
| 107 | PCA | 11.54 | 70 | 5 | 4 | 9 | pT3bNxMx | naïve | 0 | - | + | + | + |
| 108 | BPH | 8.793 | 46 |  |  |  |  |  |  |  |  |  |  |
| 109 | PCA | 6.812 | 54 | 3 | 3 | 6 | pT2xNxMx | naïve | 0 | - | + | + | + |
| 110 | BPH | 6.694 | 77 |  |  |  |  |  |  |  |  |  |  |
| 111 | PCA | 16.88 | 71 | 3 | 3 | 6 | pT2cN0Mx | naïve | 0 | - | + | + | + |
| 112 | PCA | 100 | 73 | 4 | 5 | 9 | pT2cNxMx | naïve | 0 | - | + | + | + |
| 113 | PCA | 10.52 | 66 | 3 | 3 | 6 | pT2cNxMx | naïve | 0 | - | + | + | + |
| 114 | BPH | 50.81 | 76 |  |  |  |  |  |  |  |  |  |  |
| 116 | PCA | 15.48 | 80 | 4 | 4 | 8 | pT2cNxMx | naïve | 0 | - | + | + | + |
| 117 | PCA | 36.74 | 76 | 5 | 4 | 9 | pT3aNxMx | naïve | 0 | NA | NA | NA | NA |
| 118 | PCA | 66.16 | 82 | 4 | 4 | 8 | pT3bN0Mx | naïve | 0 | NA | NA | NA | NA |
| 119 | PCA | 7.094 | 65 | 3 | 3 | 6 | pT2cNxMx | naïve | 0 | NA | NA | NA | NA |
| 120 | PCA | 6.218 | 76 | 3 | 4 | 7 | pT2cNxMx | naïve | 0 | - | + | + | + |
| 121 | BPH | 0.478 | 42 |  |  |  |  |  |  |  |  |  |  |
| 122 | PCA | 62.77 | 57 | 4 | 5 | 9 | pT3bNxM1 | naïve | 0 | - | + | + | + |
| 123 | PCA | 14.91 | 72 | 4 | 4 | 8 | pT2cNxMx | naïve | 0 | - | + | + | + |
| 124 | PCA | 15.062 | 59 | 4 | 3 | 7 | pT3aNxMx | naïve | 0 | - | + | + | + |
| 125 | PCA | 7.374 | 58 | 3 | 3 | 6 | pT3aNxMx | naïve | 0 | - | + | + | + |
| 126 | PCA | 10.337 | 55 | 4 | 5 | 9 | pT3bN0Mx | naïve | 0 | - | + | + | + |
| 127 | PCA | 100 | 81 | 4 | 4 | 8 | pT4N1Mx | naïve | 1 | - | + | + | + |
| 129 | PCA | 100 | 74 | 4 | 4 | 8 | pT2cNxMx | naïve | 0 | - | + | + | + |
| 130 | BPH | 12.2 | 59 |  |  |  |  |  |  |  |  |  |  |
| 131 | BPH | 7.94 | 44 |  |  |  |  |  |  |  |  |  |  |
| 132 | PCA | 6.189 | 72 | 4 | 3 | 7 | pT2cNxMx | naïve | 0 | - | + | + | + |
| 133 | PCA | 46.692 | 80 | 4 | 5 | 9 | pT3aN0Mx | naïve | 0 | - | + | + | + |
| 134 | PCA | 6 | 74 | 4 | 4 | 8 | pT2cN0Mx | naïve | 0 | - | + | + | + |
| 135 | BPH | 11.969 | 59 |  |  |  |  |  |  |  |  |  |  |
| 138 | BPH | 9.481 | 68 |  |  |  |  |  |  |  |  |  |  |
| 139 | PCA | 16.059 | 65 | 4 | 4 | 8 | pT3aNxMx | naïve | 0 | - | + | + | + |
| 140 | BPH | 6.79 | 61 |  |  |  |  |  |  |  |  |  |  |
| 141 | BPH | 4.75 | 59 |  |  |  |  |  |  |  |  |  |  |
| 142 | PCA | 36.47 | 63 | 5 | 5 | 10 | pT2cNxM1 | naïve | 1 | - | + | + | + |
| 143 | BPH | 6.35 | 67 |  |  |  |  |  |  |  |  |  |  |
| 144 | PCA | 10 | 72 | 3 | 3 | 6 | pT3aNxMx | naïve | 0 | - | + | + | + |
| 145 | BPH | 7.73 | 58 |  |  |  |  |  |  |  |  |  |  |
| 147 | PCA | 98.88 | 71 | 4 | 5 | 9 | pT2cNxMx | naïve | 0 | - | + | + | + |
| 148 | PCA | 15.28 | 69 | 4 | 5 | 9 | pT2aN0Mx | naïve | 0 | - | + | + | + |
| 149 | PCA | 18.2 | 59 | 4 | 5 | 9 | pT2cNxMx | naïve | 0 | - | + | + | + |
| 150 | BPH | 9.09 | 53 |  |  |  |  |  |  |  |  |  |  |
| 151 | BPH | 17.3 | 63 |  |  |  |  |  |  |  |  |  |  |
| 152 | PCA | 45.82 | 67 | 4 | 5 | 9 | pT2cNxMx | naïve | 0 | - | + | + | + |
| 153 | PCA | 100 | 72 | 4 | 4 | 8 | pT3bN0Mx | naïve | 1 | - | + | + | + |
| 154 | BPH | 10.93 | 69 |  |  |  |  |  |  |  |  |  |  |
| 155 | BPH | 7.44 | 70 |  |  |  |  |  |  |  |  |  |  |
| 156 | BPH | 5.38 | 67 |  |  |  |  |  |  |  |  |  |  |
| 157 | BPH | 18.81 | 56 |  |  |  |  |  |  |  |  |  |  |
| 158 | PCA | 45 | 69 | 5 | 5 | 10 | pT3bNxMx | naïve | 0 | - | + | + | + |
| 159 | BPH | 3.66 | 52 |  |  |  |  |  |  |  |  |  |  |
| 160 | BPH | 5.55 | 68 |  |  |  |  |  |  |  |  |  |  |
| 161 | BPH | 10.166 | 66 |  |  |  |  |  |  |  |  |  |  |
| 162 | PCA | 7.76 | 70 | 3 | 3 | 6 | pT2cNxMx | naïve | 0 | - | + | + | + |
| 163 | BPH | 7.5 | 66 |  |  |  |  |  |  |  |  |  |  |
| 164 | BPH | 8.956 | 72 |  |  |  |  |  |  |  |  |  |  |
| 165 | PCA | 9.39 | 62 | 5 | 5 | 10 | pT3bN0Mx | naïve | 0 | - | + | + | + |
| 166 | BPH | 34.9 | 55 |  |  |  |  |  |  |  |  |  |  |
| 167 | BPH | 100 | 69 |  |  |  |  |  |  |  |  |  |  |
| 168 | BPH | 6.63 | 65 |  |  |  |  |  |  |  |  |  |  |
| 169 | PCA | 9.38 | 56 | 5 | 5 | 10 | pT2aNxMz | naïve | 0 | - | + | + | + |
| 170 | PCA | 35.45 | 69 | 4 | 3 | 7 | pT4NxMx | naïve | 0 | - | + | + | + |
| 171 | PCA | 3.938 | 67 | 3 | 3 | 6 | pT2aNxMx | naïve | 0 | - | + | + | + |
| 172 | PCA | 13.86 | 71 | 5 | 5 | 10 | pT2cNxMx | naïve | 0 | - | + | + | + |
| 173 | PCA | 31.95 | 68 | 4 | 3 | 7 | pT2cN0Mx | naïve | 0 | NA | NA | NA | NA |
| 174 | BPH | 10.32 | 63 |  |  |  |  |  |  |  |  |  |  |
| 175 | PCA | 43.33 | 66 | 4 | 4 | 8 | pT3aN0Mx | naïve | 1 | NA | NA | NA | NA |
| 176 | PCA | 4.89 | 61 | 3 | 3 | 6 | pT2cNxMx | naïve | 0 | - | + | + | + |
| 177 | PCA | 20.288 | 64 | 3 | 3 | 6 | pT1bN0Mx | naïve | 0 | - | + | + | + |
| 178 | BPH | 9.491 | 59 |  |  |  |  |  |  |  |  |  |  |
| 180 | BPH | 66.02 | 68 |  |  |  |  |  |  |  |  |  |  |
| 181 | BPH | 6.12 | 68 |  |  |  |  |  |  |  |  |  |  |
| 182 | BPH | 6.4 | 59 |  |  |  |  |  |  |  |  |  |  |
| 183 | PCA | 25.76 | 73 | 3 | 4 | 7 | pT2cN0Mx | naïve | 0 | - | + | + | + |
| 184 | PCA | 5.54 | 51 | 4 | 4 | 8 | pT2aN0Mx | naïve | 0 | - | - | + | + |
| 185 | BPH | 44.698 | 69 |  |  |  |  |  |  |  |  |  |  |
| 186 | PCA | 8.26 | 61 | 3 | 4 | 7 | pT2aN0Mx | naïve | 0 | NA | NA | NA | NA |
| 187 | BPH | 7.959 | 73 |  |  |  |  |  |  |  |  |  |  |
| 188 | PCA | 10.557 | 71 | 3 | 4 | 7 | pT1aNxMx | naïve | 0 | - | + | + | + |
| 189 | BPH | 10.269 | 66 |  |  |  |  |  |  |  |  |  |  |
| 190 | BPH | 6.53 | 56 |  |  |  |  |  |  |  |  |  |  |
| 191 | PCA | 6.387 | 61 | 3 | 4 | 7 | pT1aNxMx | naïve | 0 | - | + | + | + |
| 192 | BPH | 18.91 | 73 |  |  |  |  |  |  |  |  |  |  |
| 193 | PCA | 100 | 64 | \ | \ | \ | pT2aNxMx | naïve | 0 | - | + | + | + |
| 194 | PCA | 5.737 | 76 | 3 | 4 | 7 | pT2cN0Mx | naïve | 0 | - | + | + | + |
| 195 | BPH | 5.11 | 70 |  |  |  |  |  |  |  |  |  |  |
| 196 | BPH | 6 | 69 |  |  |  |  |  |  |  |  |  |  |
| 197 | PCA | 27.45 | 74 | 3 | 4 | 7 | pT3aNxMx | naïve | 0 | - | + | + | + |
| 198 | PCA | 4.465 | 70 | 3 | 4 | 7 | pT2cNxMx | naïve | 0 | - | + | + | ± |
| 199 | PCA | 37.169 | 62 | 4 | 3 | 7 | pT2cN0Mx | naïve | 0 | - | + | + | + |
| 200 | BPH | 14.52 | 72 |  |  |  |  |  |  |  |  |  |  |
| 201 | BPH | 5.919 | 67 |  |  |  |  |  |  |  |  |  |  |
| 202 | BPH | 6.74 | 74 |  |  |  |  |  |  |  |  |  |  |
| 203 | BPH | 22.107 | 71 |  |  |  |  |  |  |  |  |  |  |
| 204 | PCA | 55.893 | 55 | 3 | 4 | 7 | pT2aNxMx | naïve | 0 | - | + | + | + |
| 205 | BPH | 10.74 | 66 |  |  |  |  |  |  |  |  |  |  |
| 206 | BPH | 4.52 | 62 |  |  |  |  |  |  |  |  |  |  |
| 207 | BPH | 6.33 | 65 |  |  |  |  |  |  |  |  |  |  |
| 208 | PCA | 100 | 60 | 4 | 4 | 8 | pT4NxMx | naïve | 0 | - | + | + | + |
| 209 | BPH | 6.48 | 50 |  |  |  |  |  |  |  |  |  |  |
| 211 | BPH | 8.27 | 49 |  |  |  |  |  |  |  |  |  |  |
| 212 | BPH | 10.34 | 56 |  |  |  |  |  |  |  |  |  |  |
| 213 | BPH | 12.73 | 64 |  |  |  |  |  |  |  |  |  |  |
| 214 | BPH | 8.16 | 61 |  |  |  |  |  |  |  |  |  |  |
| 215 | PCA | 6.33 | 69 | 3 | 4 | 7 | pT2cNxMx | naïve | 0 | - | + | + | + |
| 216 | BPH | 4.19 | 63 |  |  |  |  |  |  |  |  |  |  |
| 217 | BPH | 45.25 | 55 |  |  |  |  |  |  |  |  |  |  |
| 218 | PCA | 4.968 | 71 | 4 | 4 | 8 | pT3aN0Mx | naïve | 0 | - | + | + | + |
| 219 | PCA | 43.76 | 54 | 4 | 5 | 9 | pT2cNxMx | naïve | 0 | - | + | + | + |
| 220 | BPH | 15.438 | 64 |  |  |  |  |  |  |  |  |  |  |
| 221 | BPH | 5.591 | 74 |  |  |  |  |  |  |  |  |  |  |
| 222 | BPH | 9.5 | 54 |  |  |  |  |  |  |  |  |  |  |
| 223 | BPH | 5.76 | 63 |  |  |  |  |  |  |  |  |  |  |
| 224 | PCA | 100 | 71 | 3 | 4 | 7 | pT3bN0Mx | naïve | 0 | NA | NA | NA | NA |
| 225 | PCA | 8 | 75 | 3 | 4 | 7 | pT2aNxMx | naïve | 0 | - | + | + | + |
| 226 | PCA | 12.86 | 73 | 3 | 4 | 7 | pT2cNxMx | naïve | 0 | - | + | + | + |
| 227 | BPH | 7.22 | 72 |  |  |  |  |  |  |  |  |  |  |
| 228 | BPH | 59.036 | 59 |  |  |  |  |  |  |  |  |  |  |
| 229 | BPH | 7.97 | 61 |  |  |  |  |  |  |  |  |  |  |
| 230 | PCA | 14.93 | 77 | 5 | 4 | 9 | pT3aN1Mx | naïve | 0 | - | + | + | + |
| 231 | BPH | 9.1 | 63 |  |  |  |  |  |  |  |  |  |  |
| 232 | BPH | 6.49 | 60 |  |  |  |  |  |  |  |  |  |  |
| 233 | BPH | 5.5 | 58 |  |  |  |  |  |  |  |  |  |  |
| 234 | PCA | 100 | 51 | 4 | 4 | 8 | pT2cNxMx | naïve | 0 | - | + | + | + |
| 235 | BPH | 4.02 | 53 |  |  |  |  |  |  |  |  |  |  |
| 236 | BPH | 8.924 | 61 |  |  |  |  |  |  |  |  |  |  |
| 237 | BPH | 10.007 | 70 |  |  |  |  |  |  |  |  |  |  |
| 238 | BPH | 5.169 | 60 |  |  |  |  |  |  |  |  |  |  |
| 239 | BPH | 13.466 | 62 |  |  |  |  |  |  |  |  |  |  |
| 240 | BPH | 15.88 | 63 |  |  |  |  |  |  |  |  |  |  |
| 241 | BPH | 9 | 62 |  |  |  |  |  |  |  |  |  |  |
| 242 | BPH | 19.94 | 66 |  |  |  |  |  |  |  |  |  |  |
| 243 | BPH | 8.03 | 61 |  |  |  |  |  |  |  |  |  |  |
| 245 | BPH | 14.78 | 62 |  |  |  |  |  |  |  |  |  |  |
| 246 | BPH | 8.5 | 53 |  |  |  |  |  |  |  |  |  |  |
| 247 | BPH | 12.4 | 49 |  |  |  |  |  |  |  |  |  |  |
| 248 | PCA | 63.2 | 56 | 4 | 5 | 9 | pT2cNxMx | naïve | 0 | - | + | + | + |
| 249 | BPH | 25.9 | 57 |  |  |  |  |  |  |  |  |  |  |
| 250 | PCA | 6.288 | 60 | 3 | 3 | 6 | pT2aNxMx | naïve | 0 | - | + | + | + |
| 251 | BPH | 8.567 | 70 |  |  |  |  |  |  |  |  |  |  |
| 252 | PCA | 98.17 | 77 | 3 | 3 | 6 | pT2aN1M1 | naïve | 1 | - | + | + | + |
| 253 | PCA | 100 | 82 | 4 | 5 | 9 | pT3bN1Mx | naïve | 0 | NA | NA | NA | NA |
| 254 | PCA | 33.12 | 68 | 3 | 4 | 7 | pT2cNxMx | naïve | 0 | - | + | + | + |
| 255 | PCA | 100 | 70 | 4 | 5 | 9 | pT2cNxMx | naïve | 0 | - | + | + | + |
| 256 | BPH | 5.39 | 59 |  |  |  |  |  |  |  |  |  |  |
| 257 | BPH | 8.717 | 60 |  |  |  |  |  |  |  |  |  |  |
| 258 | BPH | 14.96 | 67 |  |  |  |  |  |  |  |  |  |  |
| 259 | PCA | 100 | 64 | 4 | 5 | 9 | pT4N1M1 | naïve | 1 | - | + | + | + |
| 260 | PCA | 12.397 | 70 | 3 | 4 | 7 | pT3bN0Mx | naïve | 0 | NA | NA | NA | NA |
| 261 | BPH | 7.37 | 61 |  |  |  |  |  |  |  |  |  |  |
| 262 | BPH | 12.46 | 59 |  |  |  |  |  |  |  |  |  |  |
| 263 | PCA | 9.1 | 68 | 4 | 4 | 8 | pT2cN0Mx | naïve | 0 | - | + | + | + |
| 265 | PCA | 43.9 | 54 | 5 | 4 | 9 | pT3aN0Mx | naïve | 0 | NA | NA | NA | NA |
| 266 | PCA | 8.93 | 87 | 5 | 4 | 9 | pT2cNxMx | Zoladex, Casodex+TURP | 0 | - | + | + | + |
| 267 | BPH | 21.677 | 73 |  |  |  |  |  |  |  |  |  |  |
| 268 | BPH | 14.188 | 71 |  |  |  |  |  |  |  |  |  |  |
| 269 | BPH | 4.56 | 51 |  |  |  |  |  |  |  |  |  |  |
| 270 | PCA | 8.69 | 75 | 4 | 5 | 9 | pT2cN0Mx | naïve | 0 | - | ± | + | + |
| 271 | BPH | 4.399 | 70 |  |  |  |  |  |  |  |  |  |  |
| 272 | BPH | 3.65 | 64 |  |  |  |  |  |  |  |  |  |  |
| 273 | BPH | 6.764 | 59 |  |  |  |  |  |  |  |  |  |  |
| 274 | PCA | 9.74 | 55 | 3 | 4 | 7 | pT2cNxMx | naïve | 0 | - | + | + | + |
| 275 | BPH | 14.59 | 54 |  |  |  |  |  |  |  |  |  |  |
| 276 | BPH | 6.517 | 71 |  |  |  |  |  |  |  |  |  |  |
| 278 | PCA | 7.572 | 66 | 3 | 3 | 6 | pT2aN0Mx | naïve | 0 | - | + | - | + |
| 279 | PCA | 100 | 63 | 4 | 5 | 9 | pT3bN0M1 | naïve | 1 | NA | NA | NA | NA |
| 280 | BPH | 6.077 | 45 |  |  |  |  |  |  |  |  |  |  |
| 281 | PCA | 24.253 | 71 | 4 | 5 | 9 | pT3bN1M1 | naïve | 1 | NA | NA | NA | NA |
| 282 | PCA | 100 | 76 | 5 | 4 | 9 | pT3bN0Mx | naïve | 0 | - | + | + | + |
| 283 | PCA | 19.49 | 80 | 3 | 4 | 7 | pT3aNxMx | naïve | 0 | - | + | + | + |
| 285 | BPH | 13.65 | 62 |  |  |  |  |  |  |  |  |  |  |
| 286 | BPH | 6.14 | 71 |  |  |  |  |  |  |  |  |  |  |
| 287 | BPH | 9.785 | 68 |  |  |  |  |  |  |  |  |  |  |
| 288 | BPH | 8.292 | 70 |  |  |  |  |  |  |  |  |  |  |
| 289 | BPH | 4.76 | 71 |  |  |  |  |  |  |  |  |  |  |
| 290 | BPH | 5.716 | 59 |  |  |  |  |  |  |  |  |  |  |
| 291 | BPH | 8.196 | 67 |  |  |  |  |  |  |  |  |  |  |
| 292 | PCA | 6.74 | 73 | 3 | 3 | 6 | pT3aNxMx | naïve | 0 | - | + | + | + |
| 293 | PCA | 43.96 | 77 | 5 | 4 | 9 | pT3bN0Mx | naïve | 0 | - | - | + | + |
| 294 | BPH | 16.43 | 56 |  |  |  |  |  |  |  |  |  |  |
| 295 | BPH | 5.94 | 65 |  |  |  |  |  |  |  |  |  |  |
| 296 | PCA | 85.78 | 60 | 4 | 5 | 9 | pT2cNxMx | naïve | 0 | - | + | + | + |
| 297 | BPH | 4.73 | 64 |  |  |  |  |  |  |  |  |  |  |
| 298 | BPH | 6.08 | 59 |  |  |  |  |  |  |  |  |  |  |
| 299 | BPH | 3.263 | 49 |  |  |  |  |  |  |  |  |  |  |
| 300 | PCA | 16.78 | 84 | 4 | 5 | 9 | pT2cNxMx | naïve | 0 | - | + | + | + |
| 301 | PCA | 61.959 | 65 | 4 | 4 | 8 | pT2cNxMx | naïve | 0 | - | + | + | + |
| 302 | PCA | 11.084 | 72 | 4 | 4 | 8 | pT3bN0Mx | naïve | 1 | - | + | + | + |
| 303 | PCA | 14.96 | 76 | 4 | 5 | 9 | pT2aNxMx | naïve | 0 | - | + | + | + |
| 304 | PCA | 36.17 | 70 | 5 | 4 | 9 | pT3bN0Mx | Bicalutamide | 0 | NA | NA | NA | NA |
| 305 | PCA | 7.572 | 66 | 3 | 3 | 6 | pT2aN0Mx | naïve | 0 | - | + | - | + |
| 306 | PCA | 23.86 | 77 | 4 | 3 | 7 | pT2cN0Mx | naïve | 0 | NA | NA | NA | NA |
| 307 | PCA | 100 | 70 | 4 | 5 | 9 | pT3bN0Mx | naïve | 0 | - | + | + | + |
| 308 | PCA | 23.63 | 72 | 4 | 5 | 9 | pT3bN1Mx | Zoladex, Casodex | 0 | - | + | + | + |
| 309 | PCA | 14.17 | 62 | 3 | 3 | 6 | pT2cN0Mx | naïve | 0 | - | + | + | + |
| 310 | PCA | 3.95 | 69 | 4 | 5 | 9 | pT2cN0Mx | naïve | 0 | NA | NA | NA | NA |
| 312 | PCA | 13.94 | 73 | 4 | 4 | 8 | pT2bNxMx | naïve | 0 | - | + | + | + |
| 313 | BPH | 6 | 60 |  |  |  |  |  |  |  |  |  |  |
| 314 | BPH | 1.59 | 73 |  |  |  |  |  |  |  |  |  |  |
| 315 | BPH | 14.188 | 71 |  |  |  |  |  |  |  |  |  |  |
| 316 | BPH | 5.011 | 76 |  |  |  |  |  |  |  |  |  |  |
| 317 | PCA | 24.253 | 71 | 4 | 5 | 9 | pT2cNxMx | naïve | 0 | - | + | + | + |
| 319 | BPH | 32.103 | 62 |  |  |  |  |  |  |  |  |  |  |
| 320 | BPH | 7.959 | 73 |  |  |  |  |  |  |  |  |  |  |
| 321 | BPH | 8.584 | 61 |  |  |  |  |  |  |  |  |  |  |
| 322 | BPH | 6.91 | 60 |  |  |  |  |  |  |  |  |  |  |
| 323 | BPH | 1.664 | 69 |  |  |  |  |  |  |  |  |  |  |
| 324 | PCA | 9.15 | 63 | 4 | 4 | 8 | pT3bNxMx | naïve | 0 | - | + | + | + |
| 325 | BPH | 5.767 | 62 |  |  |  |  |  |  |  |  |  |  |
| 326 | BPH | 5.285 | 55 |  |  |  |  |  |  |  |  |  |  |
| 329 | BPH | 23.38 | 58 |  |  |  |  |  |  |  |  |  |  |
| 330 | BPH | 8.68 | 71 |  |  |  |  |  |  |  |  |  |  |
| 331 | BPH | 5.767 | 62 |  |  |  |  |  |  |  |  |  |  |
| 332 | BPH | 5.967 | 73 |  |  |  |  |  |  |  |  |  |  |
| 333 | BPH | 19.7 | 70 |  |  |  |  |  |  |  |  |  |  |
| 334 | BPH | 58.052 | 55 |  |  |  |  |  |  |  |  |  |  |
| 335 | BPH | 13.109 | 73 |  |  |  |  |  |  |  |  |  |  |
| N291 | NC | 1.924 |  |  |  |  |  |  |  |  |  |  |  |
| N292 | NC | 0.704 |  |  |  |  |  |  |  |  |  |  |  |
| N293 | NC | 2.868 |  |  |  |  |  |  |  |  |  |  |  |
| N294 | NC | 0.088 |  |  |  |  |  |  |  |  |  |  |  |
| N295 | NC | 0.436 |  |  |  |  |  |  |  |  |  |  |  |
| N296 | NC | 2.125 |  |  |  |  |  |  |  |  |  |  |  |
| N297 | NC | 1.082 |  |  |  |  |  |  |  |  |  |  |  |
| N298 | NC | 0.913 |  |  |  |  |  |  |  |  |  |  |  |
| N299 | NC | 1.606 |  |  |  |  |  |  |  |  |  |  |  |
| N300 | NC | 1.422 |  |  |  |  |  |  |  |  |  |  |  |
| N301 | NC | 2.18 |  |  |  |  |  |  |  |  |  |  |  |
| N302 | NC | 2.334 |  |  |  |  |  |  |  |  |  |  |  |
| N307 | NC | 1.886 |  |  |  |  |  |  |  |  |  |  |  |
| N308 | NC | 2.008 |  |  |  |  |  |  |  |  |  |  |  |
| N410 | NC | 0.552 |  |  |  |  |  |  |  |  |  |  |  |
| N414 | NC | 0.356 |  |  |  |  |  |  |  |  |  |  |  |
| N415 | NC | 0.905 |  |  |  |  |  |  |  |  |  |  |  |
| N416 | NC | 1.563 |  |  |  |  |  |  |  |  |  |  |  |
| N417 | NC | 1.615 |  |  |  |  |  |  |  |  |  |  |  |
| N418 | NC | 0.092 |  |  |  |  |  |  |  |  |  |  |  |
| N419 | NC | 0.57 |  |  |  |  |  |  |  |  |  |  |  |
| N421 | NC | 1.562 |  |  |  |  |  |  |  |  |  |  |  |
| N422 | NC | 0.904 |  |  |  |  |  |  |  |  |  |  |  |
| N423 | NC | 1.541 |  |  |  |  |  |  |  |  |  |  |  |
| N424 | NC | 1.398 |  |  |  |  |  |  |  |  |  |  |  |
| N425 | NC | 0.507 |  |  |  |  |  |  |  |  |  |  |  |
| N427 | NC | 1.836 |  |  |  |  |  |  |  |  |  |  |  |
| N428 | NC | 3.008 |  |  |  |  |  |  |  |  |  |  |  |
| N429 | NC | 1.182 |  |  |  |  |  |  |  |  |  |  |  |
| N430 | NC | 0.643 |  |  |  |  |  |  |  |  |  |  |  |

|  |  |
| --- | --- |
|  |  |
|  |  |
|  |  |
|  |  |
|  |  |
|  |  |
|  |  |
|  |  |
|  |  |
|  |  |
|  |  |
|  |  |
|  |  |
|  |  |
|  |  |
|  |  |
|  |  |
|  |  |
|  |  |
|  |  |

**Additional File 1: Table S8** Demographics of PCa patients and control participants for TaqMan qPCR testing

| **NO.** | **Diagnosis** | **Diagnosis** | **PSA** | **age** | **G1** | **G2** | **Gleason score** | **Tumor stage** | **Metasatsis** | **treatment** | **CK5/6** | **P504S** | **ERG** | **PSMA** | **PSAP** |
| --- | --- | --- | --- | --- | --- | --- | --- | --- | --- | --- | --- | --- | --- | --- | --- |
| 1 | BPH | BPH | 6.91 | 59 |  |  |  |  |  |  |  |  |  |  |  |
| 2 | BPH | BPH | 6.08 | 57 |  |  |  |  |  |  |  |  |  |  |  |
| 3 | BPH | BPH | 9.76 | 69 |  |  |  |  |  |  |  |  |  |  |  |
| 4 | BPH | BPH | 131 | 40 |  |  |  |  |  |  |  |  |  |  |  |
| 5 | BPH | BPH | 13.07 | 75 |  |  |  |  |  |  |  |  |  |  |  |
| 6 | BPH | BPH | 5.767 | 62 |  |  |  |  |  |  |  |  |  |  |  |
| 7 | BPH | BPH | 8 | 74 |  |  |  |  |  |  |  |  |  |  |  |
| 8 | BPH | BPH | 5.285 | 55 |  |  |  |  |  |  |  |  |  |  |  |
| 9 | BPH | BPH | 5.967 | 73 |  |  |  |  |  |  |  |  |  |  |  |
| 10 | BPH | BPH | 19.7 | 70 |  |  |  |  |  |  |  |  |  |  |  |
| 11 | PCA | PCA | 13 | 83 | 4 | 3 | 7 | pT2cN0M1b | 1 | naïve | - | + | - | + | + |
| 12 | PCA | PCA | 4.7 | 56 | 4 | 3 | 7 | pT2cNxMx | 0 | naïve | NA | NA | NA | NA | NA |
| 13 | PCA | PCA | 12.04 | 66 | 3 | 4 | 7 | pT2cNxMx | 0 | naïve | - | + | + | + | - |
| 14 | PCA | PCA | 9.15 | 63 | 4 | 4 | 8 | pT3bNxMx | 0 | naïve | NA | + | - | + | + |
| 15 | PCA | PCA | 7.28 | 70 | 5 | 4 | 9 | pT2cNxMX | 0 | naïve | NA | NA | NA | NA | NA |
| 16 | PCA | PCA | 1.75 | 72 | 3 | 4 | 7 | pT2cNxMX | 0 | naïve | NA | NA | NA | NA | NA |
| 17 | PCA | PCA | 6.74 | 71 | 3 | 4 | 7 | pT2cN0Mx | 0 | naïve | - | + | - | + | + |
| 18 | PCA | PCA | 7.68 | 66 | 3 | 3 | 6 | pT2aNxMx | 0 | naïve | - | + | + | + | + |
| 19 | PCA | PCA | 31.59 | 57 | 4 | 3 | 7 | pT3bNxMx | 0 | naïve | - | + | - | + | ± |
| 20 | PCA | PCA | 68.52 | 66 | 3 | 4 | 7 | pT3aNxMx | 0 | naïve | NA | NA | NA | NA | NA |

Additional File 1:Table S9 Diagnosis performance of emRNAs in different PSA group

| Variables | PSA 4-10ng/ml | PSA 10-20ng/ml | PSA≥20ng/ml |
| --- | --- | --- | --- |
|  | AUC(95%CI); *P* | AUC(95%CI) ; *P* | AUC(95%CI) ; *P* |
| *CDC42* | 0.811(0.737 to 0.872)  *P* <0.0001 | 0.851(0.751 to 0.922)  *P* <0.0001 | 0.765(0.659 to 0.852)  *P* <0.0001 |
| *IL32* | 0.803(0.728 to 0.865)  *P* <0.0001 | 0.857(0.758 to 0.926)  *P* <0.0001 | 0.767(0.661 to 0.853)  *P* <0.0001 |
| *MAX* | 0.769(0.691 to 0.836)  *P* <0.0001 | 0.800(0.693 to 0.882)  *P* <0.0001 | 0.728(0.619 to 0.821)  *P* =0.0005 |
| *NCF2* | 0.763(0.685 to 0.830)  *P* <0.0001 | 0.759(0.648 to 0.849)  *P* <0.0001 | 0.678(0.566 to 0.777)  *P*=0.0275 |
| *PDGFA* | 0.788(0.711 to 0.852)  *P* <0.0001 | 0.661(0.544 to 0.765)  *P*=0.0105 | 0.582(0.468 to 0.690)  *P*= 0.2396 |
| *SRSF2* | 0.821(0.748 to 0.880)  *P* <0.0001 | 0.800(0.693 to 0.882)  *P* <0.0001 | 0.815(0.714 to 0.892)  *P* <0.0001 |
| emRNAs | 0.884(0.820-0.932)  *P*<0.001 | 0.869(0.773 to 0.935)  *P* <0.0001 | 0.835(0.737 to 0.908)  *P* <0.0001 |

Additional File 1:Table S10 Diagnosis performance of emRNAs in different ages

| Variables | AGE<55 years | AGE 55-69 years | AGE≥70 years |
| --- | --- | --- | --- |
|  | AUC(95%CI); *P* | AUC(95%CI) ; *P* | AUC(95%CI) ; *P* |
| *CDC42* | 0.783 (0.587 to 0.915)  *P* =0.0494 | 0.811(0.745 to 0.866)  *P* <0.0001 | 0.788(0.698 to 0.861)  *P* <0.0001 |
| *IL32* | 0.896 (0.721 to 0.979)  *P* <0.0001 | 0.802(0.736 to 0.858)  *P* <0.0001 | 0.821(0.735 to 0.889)  *P* <0.0001 |
| *MAX* | 0.722 (0.521 to 0.873)  *P* =0.1878 | 0.749(0.679 to 0.811)  *P* <0.0001 | 0.803(0.715 to 0.873)  *P* <0.0001 |
| *NCF2* | 0.809(0.616 to 0.932)  *P* =0.0036 | 0.715(0.642 to 0.780)  *P* <0.0001 | 0.782(0.691 to 0.856)  *P* <0.0001 |
| *PDGFA* | 0.661(0.459 to 0.828)  *P* =0.2058 | 0.733(0.661 to 0.797)  *P* <0.0001 | 0.662(0.564 to 0.751)  *P*= 0.0034 |
| *SRSF2* | 0.739 (0.540 to 0.886)  *P* =0.0457 | 0.793(0.726 to 0.851)  *P* <0.0001 | 0.794(0.705 to 0.866)  *P* <0.0001 |
| emRNAs | 0.965(0.818 to 0.999)  *P*<0.001 | 0.855(0.794 to 0.903)  *P* <0.0001 | 0.855(0.773 to 0.916)  *P* <0.0001 |

PCa: prostate cancer; AUC: area under curve; CI: confidence interval; emRNA: exosomal mRNA.

Additional File 1: Table S10 Diagnosis performance of emRNAs in agesnegative biopsy and GS 6 with GS≥7

| Variables |  |  |  |
| --- | --- | --- | --- |
|  | AUC(95%CI); *P* |  |  |
| *CDC42* | 0.757 (0.705 to 0.803)  *P* <0.0001 |  |  |
| *IL32* | 0.766 (0.715 to 0.812)  *P* <0.0001 |  |  |
| *MAX* | 0.714 (0.661 to 0.764)  *P* <0.0001 |  |  |
| *NCF2* | 0.744(0.691 to 0.791)  *P* <0.0001 |  |  |
| *PDGFA* | 0.644(0.587 to 0.697)  *P* <0.0001 |  |  |
| *SRSF2* | 0.724(0.670 to 0.773)  *P* <0.0001 |  |  |
| emRNAs | 0.788(0.738 to 0.832)  *P* <0.0001 |  |  |

PCa: prostate cancer; AUC: area under curve; CI: confidence interval; emRNA: exosomal mRNA.

**Identification the existing forms of circulating** **emRNAs**

We first answer the question of whether circulating emRNAs were existed in full-length or short variants. Kallikrein Related Peptidase 3 (*KLK3*), androgen receptor (*AR*), and Forkhead box A1 (*FOXA1*) as were selected as representative mRNAs to elucidate the existing forms of circulating emRNAs. We used Integrative Genomics Viewer (*IGV*) to visualize the reads distribution across the transcriptome of *KLK3*, *AR*, *FOXA1* in exosomes and tissue. After comparing the expression levels of each exon of exosomal *KLK3* with that of tissue *KLK3*, we found that there were more short variants in exosomal *KLK3* than in tissue *KLK3* (**Additional File 1: Figure S2a**). In order to confirm this result, we used RT-PCR to amplify the whole transcripts of *KLK3*, *AR* and *FOXA1* (primers are listed in **Additional File 1: Table S2**). As shown in **Additional File 1: Figure S2b**, *KLK3* and *AR* could not be detected in exosomes. Although the whole transcript of *FOXA1* was detectable in exosomes, the signal was quite weaker than that in tissues and cell lines. We further used qPCR to evaluate the expression levels of the variants of exosomal *KLK3*. Multiple primers for different regions in the transcript of *KLK3* were designed (primers are listed in **Additional File 1: Table S2**). As shown in **Additional File 1: Figure S2c**, the expression levels of the amplicons of *KLK3* were quite different. The same findings were confirmed in 13 PCa-associated emRNAs (*TXK, ATM, TOX4, MAX, STK4, GRK5, PDGFA, RASSF5, IL32, CDC42, FAM228B, NCF2* and *SRSF2*). As shown in **Additional File 1: Figure S4b**, more variants were predicted by IGV in exosomes than in tissue. In addition, RT-PCR results confirmed that different expression levels were found among multiple amplicons of each single emRNA (**Additional File 1: Figure S4c**). These results demonstrated that emRNAs were existing in the form of variants with different expression levels in circulation.

**Optimized detection strategy for the detection of 13 PCa-associated emRNAs**

The work-flow was summarized in **Additional File 1: Figure S4a**, including identifying emRNA candidates by RNA sequencing, visualizing the reads distribution of emRNAs by IGV, validating the primers by RT-PCR, and designing qPCR probes for emRNA detection. Specifically, we first identified 13 emRNA candidates (*TXK*, *ATM*, *TOX4*, *MAX*, *STK4*, *GRK5*, *PDGFA*, *RASSF5*, *IL32*, *CDC42*, *FAM228B*, *NCF2* and *SRSF2*) which were associated with PCa by RNA sequencing. We then visualized the read density of each candidate emRNA across the transcriptome by IGV (**Additional File 1: Figure S4b**). According to the reads distribution of emRNAs, we designed multiple primers for different regions in the transcript of each emRNA and validated the primers by reverse transcription polymerase chain reaction (RT-PCR) (primers are listed in **Additional File 1: Table S2**). Then, the detectable and unique bands were chosen as the targeted amplicon to design qPCR probes for emRNA detection (**Additional File 1: Figure S4c**).

**Established the subtype signatures for the detection of PCa**

The subtype signatures for the detection of PCa were established based on PSA, age and Gleason score (GS) (**Additional File 1: Figure S8**). Based on PSA levels, the patients were divided into 3 subgroups, PSA <10ng/ml, PSA 10-20ng/ml and PSA >20ng/ml. ROC analysis showed that *CDC42*, *IL32*, *MAX*, *NCF2* and *SRSF2* achieved good performance for PCa diagnosis in all subgroups and *PDGFA* achieved good performance in PSA <10ng/ml and PSA 10-20ng/ml groups and moderate performance in PSA >20ng/ml group (**Additional File 1: Figure S8a-b**, the detailed information was summarized in **Additional File 1: Table S9**). EmRNA-based signatures for PCa diagnosis in PSA-based subgroups were established by logistic regression analysis. ROC analysis showed that these signatures yielded an AUC of 0.884 in PSA <10ng/ml group, 0.869 in PSA 10-20ng/ml, and 0.835 in PSA >20ng/ml group (the detailed information was summarized in **Additional File 1: Table S9**).

Based on age, the patients were divided into 3 subgroups, <55 years, 55-69 years and >70 years. ROC analysis showed that all 6 emRNAs achieved good performance for PCa diagnosis in all subgroups (**Additional File 1: Figure S8c-d**, the detailed information was summarized in **Additional File 1: Table S10**). EmRNA-based signatures for PCa diagnosis in age-based subgroups were established by logistic regression analysis. ROC analysis showed that these signatures yielded an AUC of 0.965 in <55 years group, 0.855 in 55-69 years group, and 0.855 in >70 years group (the detailed information was summarized in **Additional File 1: Table S10**).

In order to distinguish the clinically significant PCa, we divided the patients into 2 subgroups, high-grade (HG) PCa (≥GS7) and BPH+ low-grade (LG) PCa (GS6). ROC analysis showed that all 6 emRNAs were upregulated in HGPCa compared to BPH plus LGPCa and achieved good performance for HGPCa diagnosis (**Additional File 1: Figure S8f-g**, the detailed information was summarized in **Additional File 1: Table S11**). EmRNA-based signatures for HGPCa diagnosis were established by logistic regression analysis and achieved a good diagnostic performance for the detection of HGPCa (AUC = 0.788).

**Correlation analysis** **between emRNAs and the grade of PCa aggressiveness**

We compared the expression levels of the emRNAs among PCa with low (GS6), intermediate (GS7) and high (GS≥8) GS samples. As shown in our results (**Additional File 1: Figure S8e**), there was no significant difference comparing the expression levels of these emRNAs among the three groups, indicating that none of 6 emRNAs was correlated with the degree of tumor aggressiveness.

**The source of circulating emRNAs.**

RNA-seq was applied to evaluate and compare the expression levels of total mRNAs or oncogene mRNAs between PCa cell lines and exosomes from the cell culture medium, as well as exosomes from the blood of human PCa xenografts and controls. We found that both total mRNAs (r = 0.449 for PC3 and r = 0.5375 for C4-2B, **Additional File 1: Figure S9a**) and oncogene mRNAs (r = 0.4354 for PC3 and r = 0.6879 for C4-2B, **Additional File 1: Figure S9b**) in exosomes from PC3 or C4-2B cell culture medium were correlated with those from each PCa cell line. Additionally, the heatmap showed that the most dysregulated mRNAs in PCa or C4-2B cells compared to these mRNAs in control RWPE-1 cells presented the same expression tendency in exosomes from PCa cell culture medium compared to control cell culture medium (**Additional File 1: Figure S9d**). Furthermore, the mRNA expression levels in exosomes from the blood of human PCa xenografts were significantly correlated with those in exosomes from control blood (r = 0.546, **Additional File 1: Figure S9c**), indicating that the PCa xenograft could pack mRNA into exosomes and further release them into the circulation in mice. To further verify that PCa-associated emRNAs are derived from PCa, we evaluated 6 representative PCa-associated circulating emRNAs (*CDC42*, *IL32*, *MAX*, *NCF2*, *PDGFA* and *SRSF2*) in PCa patients (n = 6) before and 1 month after prostatectomy. The expression levels of all representative emRNAs were found to decrease significantly after surgery (**Additional File 1: Figure S9e**). Therefore, our results indicated that PCa cells could pack emRNAs and then release them into the cell culture medium or circulation.

**The potential importance of the emRNAs**

It was suggested that emRNAs may act as potential biomarkers for cancer diagnosis, such as glioma[1] and pancreatic ductal adenocarcinoma (PDAC) [2], however, there is currently no comprehensive study on systematically evaluating the role of circulating emRNAs in PCa screening and diagnosis. In our study, we identified PCa associated emRNAs and established emRNA-based signatures for PCa screening and diagnosis. Currently, the diagnosis of PCa in men with moderately elevated PSA is challenging. The efficacy of novel biomarkers such as PCA3 (AUC 0.64 to 0.76, with AUC increments over PSA 0 to 0.16) [3], and PHI (AUC 0.703, with AUC increment over PSA of 0.178) [4] are still controversial. Our diagnostic signature demonstrated superior diagnostic accuracy to PSA and could avoid unnecessary prostatic biopsies. In this context, our study may provide new insights into the role of emRNAs in diagnosing PCa.

Furthermore, we performed KEGG pathway enrichment analysis, which demonstrated that the most relevant pathways in PCa circulating exosomes were the PI3K-Akt and MAPK signaling pathways (**Additional File 1: Figure S10a**). The key emRNAs identified by the molecular network of the relevant pathways, including ECM-receptor interaction and EGFR tyrosine kinase inhibitor resistance, was predicted to play major roles in PCa pathogenesis, providing future targets for mechanistic studies (**Additional File 1: Figure S10b**). Investigation of these relevant pathways and their key mRNAs in circulating exosomes could help to clarify mechanisms related to PCa progression, especially distant metastasis. Circulating emRNAs could not only serve as novel biomarkers for PCa diagnosis but also play a significant role in PCa progression.

**Methods**

**Study design**

The work-flow of the study was summarized in **Fig. 1a**, including sample processing, emRNA sequencing, demonstrating the landscapes of emRNA, optimizing the detection strategy, and identifying tumor-specific emRNA signatures. Specifically, we first isolated the exosomes from serum samples. The exosome RNAs were then purified. We next sequenced the strand-specific RNA-seq libraries. We further illustrated the landscape and characteristics of circulating emRNAs. Based on the existing forms of emRNAs in circulating exosomes, we developed an improved strategy for emRNA detection. The new detection strategy was applied to evaluate the expression of emRNAs candidates for PCa detection. Finally, novel emRNA signatures for PCa screening and diagnosis were established and the diagnostic performance of emRNA signatures was evaluated.

**Sample collection and processing**

This project was approved by the Clinical Research Ethics Committee of Shanghai Changhai Hospital of Second Military Medical University (no. NSFC81430058). All of the clinical samples were obtained from Shanghai Changhai Hospital (Shanghai, China). Written informed consents were obtained from the participants before sampling.

Blood samples were collected from patients undergoing prostate biopsy from January 2016 to November 2018. PCa and BPH samples were confirmed by prostate biopsy and the pathology of the biopsy tissues was examined by two pathologists to confirm the diagnosis and Gleason score. Patients who underwent biopsy were assessed to have a PSA increase greater than 4 ng/ml or PSA that was not elevated, but the rectal examination revealed a nodule or imaging examination abnormality. On the first day of admission, each patient signed an informed consent form, the label was printed and attached to the BD serum collection tube. On the second morning of admission, 3-5 ml of fasting peripheral blood was collected from the patient. Samples were stored at 4 °C, transported to the laboratory with ice to separate the serum, centrifuged at 1500 xg for 20 minutes, and the supernatant was collected. The supernatant was placed in a 1.5 ml centrifuge tube and numbered with the admission number and specimen type. Exosomes were immediately isolated after collection or stored at -80 °C until further processing. Demographics of the PCa patients and control participants for QC of exosome isolation are summarized in **Additional File 1: Table S5**. Demographics of the PCa patients and control participants for RNA-seq of their circulating exosomes are summarized in **Additional File 1: Table S6**. Demographics of the PCa patients and control participants for dysregulated mRNA validation are summarized in **Additional File 1: Table S7**. Demographics of 10 pairs of PCa patients and controls are summarized in **Additional File 1: Table S8**)

PCa and adjacent normal tissues were collected from patients who underwent radical prostatectomy at Shanghai Changhai Hospital. The hematoxylin and eosin (H&E) stained slides of tumor tissues were examined by two pathologists to confirm the diagnosis and Gleason score. Total RNA was extracted immediately after collection or stored at -80 °C until further processing.

**Exosome isolation**

The exoEasy Maxi Kit (Qiagen 76064) [5] was used to extract exosomes. Five hundred microliters of serum were isolated by standard operation. If the serum had been stored at -80 °C, it was placed on ice and slowly thawed and then incubated at room temperature until completely thawed. Serum exosomes were extracted according to the manufacturer’s instructions. First, serum was filtered using a 0.22 μm (Millipore) filter, and then 450 μl of the filtered serum was mixed with an equal volume of XBP buffer. After mixing, 900 μl of the solution was added to the adsorption column and then centrifuged at 500 xg for 1 minute at room temperature, which was repeated one more time. The flow-through solution from the adsorption column was discarded, and the serum exosomes were immobilized on the adsorption column membrane. Then, 5 ml of XWP buffer was added to the adsorption column and centrifuged at 5000 xg for 5 minutes at room temperature. The cells were incubated with 400 μl of XE buffer for 1 minute and centrifuged at 500 xg for 5 minutes. The eluate was re-added to the exoEasy spin column membrane and incubated for 1 minute, centrifuged at 5000 xg for 5 minutes to collect the eluate and transferred to a 1.5 ml centrifuge tube. Exosomes could be used for further research or stored at -80 °C.

**Electron Microscopy**

The purified exosome sample was diluted 1000-fold with PBS (HyClone), filtered through a 0.22 μm filter membrane (Millipore) and used for electron microscopy (JEOL). Twenty microliters of the diluted sample were pipetted, dropped onto a copper mesh, and allowed to stand for 20 minutes, and then the liquid was blotted with filter paper to prepare the negative stain. Negative staining was performed with 1% phosphotungstic acid for approximately 3 seconds, filter paper was used to blot the excess liquid, and the exosomes were identified by electron microscopy.

**Nanoparticle Tracking Analysis**

The extracted exosomes were diluted 1:200 (filtered PBS). The module detected by the Nano Sight 300 (Malvern) was washed with ddH2O. The diluted exosomes were sucked into the syringe, placed on the electric pump, and connected to the detection module. The provided software was used to create a new file, adjust the focus and exposure intensity, select the appropriate field of view, start detection, record for 30 s, and calculate and export the results.

**Western blotting**

A total of 50 μL RIPA lysis buffer (Thermo Fisher) was added to the extracted exosomes. Then, 0.5 μL of Protease Inhibitor (Bimake) was added, and the mixture was centrifuged at 12000 xg for 15 minutes at 4 °C. The supernatant was collected, and the concentration was measured using a BCA kit (Thermo Fisher). Then, 5X loading buffer (Sangon) was added, and the samples was incubated in a 100 °C metal bath (Eppendorf) for 15 min. A PAGE Gel Rapid Preparation Kit (EpiZyme) was used to make a 10% gel according to the manufacturer’s instructions. Then, 30-50 μg exosome protein samples were added to each well. Protein marker (Bio-Rad) was added to the blank well. The machine (Bio-Rad) was set to 100 V, and electrophoresis was performed for 2 hours. After electrophoresis was completed, the gel was removed, stacked with a PVDF (Millipore) membrane, placed in a transfer tank, and filled with transfer buffer. The instrument was set to 100 V for 1.5 hours. After the transfer was completed, the membranes were blocked with 5% BSA for 2 hours at room temperature, and the membranes were cut at appropriate positions. Then, antibodies against CD9 (1:1000; ABGENT), CD63 (1:1000; ABGENT) TSG101 (1:2000; ABGENT), β-actin (1:10000; Sigma) and GAPDH (1:20000; Sigma) were added and incubated at 4 °C overnight. The next day, the membranes were placed at room temperature for 15 minutes, and the primary antibody was removed. The membranes were washed 3 times with TBST (Sangon) for 5 minutes each time. Then, secondary antibodies were added, incubated for 2 hours at room temperature, and washed 3 times with TBST for 5 minutes each time. Clarity Max Western ECL Substrate (Bio-Rad) was added. Blots were imaged by AI600 (GE).

**Circulating Exosomal RNA Purification and Sequencing**

Circulating exosome RNA was purified using an exoRNeasy Serum/Plasma Maxi Kit (Qiagen 77064). PolyA+ RNA was purified with hybridization to oligo (dT) beads (NEB). Strand-specific RNA-seq libraries were prepared using the NEBNext Ultra II Directional RNA Library Prep Kit for Illumina (NEB). Briefly, the entire exosomal RNA samples (approximately 10 ng) were fragmented and then used for first- and second-strand cDNA synthesis with random hexamer primers. For second-strand cDNA synthesis, a dUTP mix was used, which allows for the removal of the second strand. After purification of the double-stranded cDNA, the cDNA fragments were treated to repair the ends and finally ligated to adapters. Ligated cDNA products were purified and amplified by PCR with 15 cycles. The library quality was determined by a Bioanalyzer 2100 (Agilent). The strand-specific RNA-seq libraries were sequenced using the HiSeq X10 platform. Similar sequencing depths were achieved for all samples (10 Gb raw data for cells, cell-derived exosomes, PCa and BPH serum exosomes).

**Sequencing data processing**

Sequencing reads were mapped to the reference human genome (hg38) by Hisat2 after quality control processing was conducted by trimming Illumina adaptors, low-quality ends from reads with a cutoff of 20, and reads that became shorter than 20 bp using TrimGalore. Finally, we used Stringtie for expression quantification, and the R package Deseq2 was used to identify the differentially expressed genes between different groups. The gene annotation database was GENCODE V30.

**RNA purification and reverse transcription**

RNA was purified from the cells and tissues using the HiPure Total RNA Mini kit (Magen) following the manufacturer's instructions. The circulating exosomal RNA was purified using an exoRNeasy Serum/Plasma Maxi Kit (Qiagen 77064). RNA was reverse transcribed into cDNA according to the instructions of the PrimeScriptTM II 1st Strand cDNA Synthesis Kit (Takara). Fifteen microliters of mRNA in exosomes, 1 μl of random 6-mer oligos and 1 μl of 10 mM dNTPs were mixed, placed at 65 °C for approximately 5 minutes and quickly placed on ice for cooling. Five microliters of 5x PrimeScript II buffer, 0.5 μl of RNase inhibitor, 1 μl of reverse transcriptase, and RNase-free water to bring the reaction volume to 25 μl were added to the solution. The reverse transcription reaction conditions were as follows: 30 °C for 10 minutes, 42 °C for 60 minutes, 95 °C for 5 minutes, and cooling on ice for 5 minutes. The synthesized cDNA could be used immediately for subsequent qPCR reactions or temporarily stored at -20 °C.

**DNA gel electrophoresis**

Validation of PCR products was performed using DNA gel electrophoresis. First, 30 ml TAE and 0.3 g solid agarose were mixed and heated in a microwave at 66% power for 2 minutes. Disposable cups were used to prevent evaporation the nucleic acid stain Gel-Red (1:10000, Biotium) was added, and the gel was poured when the solution was approximately 70 °C. The solidified gel was placed in a nucleic acid electrophoresis tank, And the samples were loaded. The electrophoresis conditions were typically set to 100 V, 400 mA, and 40 min. After running, an image of the gel was obtained.

**Real-time Quantitative PCR**

The primers and probes are summarized in **Additional File 1: Table S2**. An ABI 7500 fluorescent PCR instrument (GE) was used. Using 20 μl of the qPCR reaction system (Thermo Fisher), specifically 10 μl of 2×qPCR Mix and 2 μl of cDNA solution, the working concentration of the upstream and downstream primers of the external reference was 200 nM, and the working concentration of the specific target upstream and downstream primers was 200 nM. The probes were used at a concentration of 100 nM, and the reaction was brought up to 20 μl with RNase-free water. The qPCR reaction procedure was set as follows: 37 °C for 5 minutes, 95 °C for 10 minutes, and 40 cycles of 95 °C for 15 seconds and extension at 60 °C for 60 seconds. The fluorescent signals were recorded during the extension phase. During the reaction, two specific targets were detected in each reaction well, the fluorescence group was FAM and VIC, for the external reference target the fluorescence group was CY5, and all the probe quenching groups were MGB. Each experiment was repeated three times. In the data processing, the mean of the Ct value of the repeated experiment was used to represent the relative expression level of the target in the sample. In each qPCR reaction plate, RNase-free water was used as a negative control, and cDNA obtained by reverse transcription of total RNA from the RWPE-1 cell line was used as a positive control.

**EmRNA quantification**

The detection of mRNAs in tissue depends on internal reference genes such as *ACTB* and *GAPDH*. However, current studies have provided no well-recognized internal reference transcripts for the detection of emRNAs. Previous studies have used *GAPDH* (glyceraldehyde-3-phosphate dehydrogenase) [6], *GAPDH/ACTB* (actin beta) [7] and *SPDEF* [8] (SAM pointed domain containing ETS transcription factor) as endogenous reference genes for circulating exosomal mRNA. Nevertheless, there is still no evidence to demonstrate their stability and reliability. In this study, we first demonstrated whether these previous reference genes were suitable for emRNA detection (**Additional File 1: Figure S6**). The workflow was summarized in **Additional File 1: Figure S6a**. The expression levels of these genes between PCa and control were shown in **Additional File 1: Figure S6b-d** and the reads distribution was shown in **Additional File 1: Figure S6e-g**. Our results showed that *GAPDH* was not detected in circulating exosomes even though several primers were designed for RT-PCR (**Additional File 1: Figure S6h**). Although *ACTB* was detectable in circulating exosomes (**Additional File 1: Figure S6**), it was found to vary substantially in different patients (**Additional File 1: Figure S6j-k**). *SPDEF*, used as an internal control for urinary emRNAs quantification, was not suitable for circulating emRNAs detection due to very low expression in RNA-seq (**Additional File 1: Figure S6c**). Thus, we applied the standard-curve quantitation method for emRNA quantification. This method is similar to previous approaches used for other RNA forms [9]. Briefly, we synthesized the amplified fragments of each target gene. During the reverse transcription of these genes, we generated a standard curve using real-time quantitative PCR by testing synthesized transcripts at different copy number concentration gradients. By this means, we could calculate the copy numbers of target genes relative to a standard sample. Specifically, the initial amount of 400ul serum was defined to extract RNA from exosomes. Then, the extracted RNA was dissolved in RNase-free water. Then, 20 µl of emRNA was processed for reverse transcription to yield 60 µl of cDNA. Then, we added 2 µl of cDNA to 20 µl of the fluorescence PCR system, with triplicate samples accessed to yield the mean CT value. At the same time, we synthesized the target RNA transcripts at copy numbers of 103, 104, 105, 106 and 107. These synthesized mRNAs were processed using the same procedure as the abovementioned transcripts (20 µl emRNA to 60 µl cDNA and 2 µl cDNA to 20 µl PCR system). These synthesized mRNAs yielded a standard curve, and we could calculate the copy number of the target emRNA by matching the CT value to the standard curve (**Additional File 1: Figure S7**).

**Data availability**

Circulating exosomal RNA sequencing data of PCa and RCC were deposited into CNGB Sequence Archive (CNSA: https://db.cngb.org/cnsa/) of CNGBdb with accession number CNP0000926.

**Cell lines and mice**

The prostate cancer cell lines C4-2B (provided by Leland Chung, Cedars-Sinai Medical Center), LNCaP (ATCC), C4-2 (ATCC) and PC-3 (ATCC) were cultured in RPMI 1640 medium (Gibco) with 10% FBS (fetal bovine serum, Gibco) and 1% antibiotic-antimycotic (Gibco). The normal prostate epithelial cell line RWPE-1 was maintained in K-SFM medium (Gibco). All cells were cultured in a 5% CO2, 37 °C incubator. Animal experiments were approved by the Laboratory Animal Ethics Committee of the Second Military Medical University (SYXK(SH)2017-0004) with the Guidelines for Animal Health and Use (Ministry of Science and Technology, China, 2006). PC-3 cells were suspended in a solution of 50% Matrigel (BD) in Hank’s Balanced Salt Solution (HBSS) (Gibco) and injected into 6-week-old male athymic nude mice. After 6 weeks, the mice were euthanized, and their tumor tissues and blood were collected.

**Statistics**

Data analysis was performed using SPSS software v21.0 (IBM), Med calc v13.0 (MedCalc Software bvba) and R software v3.5.0. The Lasso regression method was used to perform regression analysis on the sequencing results. The t-test (Student's t-test) was used for the measurement data with normally distributed continuous variables, and the Mann-Whitney U test method was used for variables with no specific distribution. The count data were compared using the Pearson chi-square test or Fisher's test to compare the differences between two groups. Univariate risk factors for PCa were calculated using one-way logistic regression. Multivariate regression analysis was used to establish the PCa screening and diagnostic signatures. The ROC (receiver operating characteristic) curve and the area under the curve (AUC) were used to evaluate the specificity and sensitivity of the indicators.

**Reference**

1.

2.

3.

4.

5.

1. Wei Z, Batagov AO, Schinelli S, Wang J, Wang Y, El Fatimy R, Rabinovsky R, Balaj L, Chen CC, Hochberg F, et al: **Coding and noncoding landscape of extracellular RNA released by human glioma stem cells.** *Nat Commun* 2017, **8:**1145.

2. Yu S, Li Y, Liao Z, Wang Z, Wang Z, Li Y, Qian L, Zhao J, Zong H, Kang B, et al: **Plasma extracellular vesicle long RNA profiling identifies a diagnostic signature for the detection of pancreatic ductal adenocarcinoma.** *Gut* 2020, **69:**540-550.

3. Auprich M, Bjartell A, Chun FK, de la Taille A, Freedland SJ, Haese A, Schalken J, Stenzl A, Tombal B, van der Poel H: **Contemporary role of prostate cancer antigen 3 in the management of prostate cancer.** *Eur Urol* 2011, **60:**1045-1054.

4. Catalona WJ, Partin AW, Sanda MG, Wei JT, Klee GG, Bangma CH, Slawin KM, Marks LS, Loeb S, Broyles DL, et al: **A multicenter study of [-2]pro-prostate specific antigen combined with prostate specific antigen and free prostate specific antigen for prostate cancer detection in the 2.0 to 10.0 ng/ml prostate specific antigen range.** *J Urol* 2011, **185:**1650-1655.

5. Enderle D, Spiel A, Coticchia CM, Berghoff E, Mueller R, Schlumpberger M, Sprenger-Haussels M, Shaffer JM, Lader E, Skog J, Noerholm M: **Characterization of RNA from Exosomes and Other Extracellular Vesicles Isolated by a Novel Spin Column-Based Method.** *PLoS One* 2015, **10:**e0136133.

6. Xu H, Chen Y, Dong X, Wang X: **Serum Exosomal Long Noncoding RNAs ENSG00000258332.1 and LINC00635 for the Diagnosis and Prognosis of Hepatocellular Carcinoma.** *Cancer Epidemiol Biomarkers Prev* 2018, **27:**710-716.

7. Li F, Yoshizawa JM, Kim KM, Kanjanapangka J, Grogan TR, Wang X, Elashoff DE, Ishikawa S, Chia D, Liao W, et al: **Discovery and Validation of Salivary Extracellular RNA Biomarkers for Noninvasive Detection of Gastric Cancer.** *Clin Chem* 2018, **64:**1513-1521.

8. McKiernan J, Donovan MJ, O'Neill V, Bentink S, Noerholm M, Belzer S, Skog J, Kattan MW, Partin A, Andriole G, et al: **A Novel Urine Exosome Gene Expression Assay to Predict High-grade Prostate Cancer at Initial Biopsy.** *JAMA Oncol* 2016, **2:**882-889.

9. Li Y, Elashoff D, Oh M, Sinha U, St John MA, Zhou X, Abemayor E, Wong DT: **Serum circulating human mRNA profiling and its utility for oral cancer detection.** *J Clin Oncol* 2006, **24:**1754-1760.
